# Heat Stress Tolerance in Rice (*Oryza sativa* L.): Identification of Quantitative Trait Loci and Candidate Genes for Seedling Growth Under Heat Stress

**DOI:** 10.3389/fpls.2018.01578

**Published:** 2018-11-01

**Authors:** Newton Lwiyiso Kilasi, Jugpreet Singh, Carlos Eduardo Vallejos, Changrong Ye, S. V. Krishna Jagadish, Paul Kusolwa, Bala Rathinasabapathi

**Affiliations:** ^1^Horticultural Sciences Department, University of Florida, Gainesville, FL, United States; ^2^Department of Crop Science and Production, Sokoine University of Agriculture, Morogoro, Tanzania; ^3^Institute of Food Crops, Yunnan Academy of Agricultural Sciences, Kunming, China; ^4^Department of Agronomy, Kansas State University, Manhattan, KS, United States

**Keywords:** aus, genotyping-by-sequencing, Nagina 22, quantitative trait loci, root growth, shoot growth

## Abstract

Productivity of rice, world's most important cereal is threatened by high temperature stress, intensified by climate change. Development of heat stress-tolerant varieties is one of the best strategies to maintain its productivity. However, heat stress tolerance is a multigenic trait and the candidate genes are poorly known. Therefore, we aimed to identify quantitative trait loci (QTL) for vegetative stage tolerance to heat stress in rice and the corresponding candidate genes. We used genotyping-by-sequencing to generate single nucleotide polymorphic (SNP) markers and genotype 150 F_8_ recombinant inbred lines (RILs) obtained by crossing heat tolerant “N22” and heat susceptible “IR64” varieties. A linkage map was constructed using 4,074 high quality SNP markers that corresponded to 1,638 recombinationally unique events in this mapping population. Six QTL for root length and two for shoot length under control conditions with 2.1–12% effect were identified. One QTL *rlht5.1* was identified for “root length under heat stress,” with 20.4% effect. Four QTL were identified for “root length under heat stress as percent of control” that explained the total phenotypic variation from 5.2 to 8.6%. Three QTL with 5.3–10.2% effect were identified for “shoot length under heat stress,” and seven QTL with 6.6–19% effect were identified for “shoot length under heat stress expressed as percentage of control.” Among the QTL identified six were overlapping between those identified using shoot traits and root traits: two were overlapping between QTL identified for “shoot length under heat stress” and “root length expressed as percentage of control” and two QTL for “shoot length as percentage of control” were overlapping a QTL each for “root length as percentage of control” and “shoot length under heat stress.” Genes coding 1,037 potential transcripts were identified based on their location in 10 QTL regions for vegetative stage heat stress tolerance. Among these, 213 transcript annotations were reported to be connected to stress tolerance in previous research in the literature. These putative candidate genes included transcription factors, chaperone proteins (e.g., alpha-crystallin family heat shock protein 20 and DNAJ homolog heat shock protein), proteases, protein kinases, phospholipases, and proteins related to disease resistance and defense and several novel proteins currently annotated as expressed and hypothetical proteins.

## Introduction

World population growth necessitates our best efforts to increase crop production by 50% before 2030 (Tomlinson, 2013). However, adding to the challenge is the projected global climate change which is expected to have negative impacts on agricultural productivity through higher than optimal temperatures for crop production (Jagadish et al., 2012). By one estimate, average temperature is expected to rise by 2–3°C over the next 30–50 years (Hatfield and Prueger, 2015). In this context, genetic improvement of heat stress tolerance traits of staple crops is of immediate necessity.

Worldwide rice (*Oryza sativa* L.) is the most widely consumed food crop. Diurnal temperature fluctuations in most rice producing areas are close to the optimum: 28 and 22°C day and night mean temperatures, respectively (Das et al., 2014). Although rice can still maintain normal growth at temperatures ranging from 27 to 32°C without significant reduction in grain yield (Aghamolki et al., 2014), temperatures above 32°C negatively affect all stages of rice plant growth and development (Aghamolki et al., 2014): the most critical temperature was found to be 33°C during the flowering stage (Jagadish et al., 2007). High temperature is detrimental to most physiological processes including stomatal opening, photosynthesis, growth, and grain yield. Heat tolerance studies in rice have mainly focused on the reproductive stage due to its high sensitivity and its immediate relevance to grain yield (Prasad et al., 2008; Jagadish et al., 2010b; Aghamolki et al., 2014; Das et al., 2014; Hatfield and Prueger, 2015). For instance, exposure of “IR64” spikelet tissue to temperatures above 29.6°C for up to 1 h during anthesis caused a reduction in spikelet fertility by 7%, per 1°C increase (Jagadish et al., 2007). Heat stress results in poor pollen viability (Ye et al., 2015a). Similarly, the efficiency of pollen production and anther dehiscence in rice varieties grown under heat stress have been shown to be reduced, thus compromising the number of pollen produced and pollen shedding (Prasad et al., 2006). Most of these studies have utilized the available natural genetic variation to study the physiology and genetics of heat stress tolerance in rice at the reproductive stage (Prasad et al., 2006; Jagadish and Pal, 2009; Jagadish et al., 2010a,b, 2013; Tenorio et al., 2013; Bahuguna et al., 2015; Glaubitz et al., 2015; Shi et al., 2015). The *aus indica* type rice variety “Nagina22” (“N22”) has been characterized for several heat stress tolerant traits (Jagadish et al., 2007, 2008; Ye et al., 2012, 2015a; Bahuguna et al., 2015). This variety has some undesirable traits like relatively small-sized grains and weak and long stems, a trait that leads to lodging of the plant and loss of grain yield (Bahuguna et al., 2015). “N22” also carries some desirable morphological and physiological characters like early maturity, high capacity for regeneration and recovery processes, and flexibility in the accumulation and mobilization of carbohydrates (Gorantla et al., 2007). However, tolerance to heat stress is a prominent trait associated with this variety that can be leveraged to identify genetic regulation of heat stress tolerance in rice. Moreover, the heat-stress tolerant traits from “N22” can be used for introgression into other varieties for developing climate-change ready rice (Ye et al., 2012). A previous study has identified quantitative trait loci (QTL) for heat stress tolerance during the reproductive stage in rice by using a recombinant inbred population between “N22” and “IR64” (Ye et al., 2012). This study further led to fine-mapping one of the identified QTL (Ye et al., 2015a,b), but the candidate genes for heat tolerance have not been identified. A metabolomic and transcriptomic study focused on the floral organs implicated genes related to sugar supply being differently expressed between “N22” and heat stress sensitive variety “Moroberekan” (Li et al., 2015). These studies focused on reproductive stage, raise further questions about the genetic control of heat stress tolerance during other developmental stages in rice. It might be possible that different genes act during different developmental stages to confer tolerance to heat stress.

Our work has therefore focused on understanding the genetics of heat-stress tolerance during early seedling growth and development in rice. Early seedling stage heat stress tolerance is critical for crop establishment especially under direct seeded conditions and could also be connected to heat stress tolerance in other stages of crop growth. Hence, selection of heat-tolerant seedlings can play a critical role in improving the efficiency for breeding stress tolerant rice varieties. We used a recombinant inbred population between “N22” (heat tolerant) and “IR64” (heat susceptible) to screen root and shoot growth traits during early seedling development under control and stress conditions. The genotyping-by-sequencing approach (GBS), (Elshire et al., 2011) was used to develop single nucleotide polymorphism (SNP) markers for genotyping and linkage map construction. Here we report on the identification of 11 QTL that control young seedling tolerance to heat stress. By further analyzing the chromosomal position of SNP spanning the QTL regions, we propose multiple candidate genes related to heat-stress tolerance during early seedling growth in rice.

## Materials and methods

### Plant materials

This research used recombinant inbred lines (RILs) generated by the International Rice Research Institute (IRRI) from a cross between “N22” and “IR64” rice varieties. Seeds of these lines (F_7_) were transported to the University of Florida under a material exchange agreement and USDA APHIS permit (PCIP-13-00281).

### Methods

To increase the seeds, four seeds from each line were dehusked, surface-sterilized with 50% (v/v) of commercial bleach, and rinsed in sterile water. The seeds were germinated in wet paper towels for 4 days and planted in potting medium (Fafard 2, Sun Gro Horticulture Canada Ltd., Canada) supplemented with 0.5 g/L Sprint 330 (Becker Underwood Inc., Ames, IA, USA) in 3-gallon containers. The germinated seedlings were planted in a greenhouse in Gainesville, Florida, in May 2014 and harvested in September that year. During this period, the average temperature of the greenhouse recorded at noon was 31°C. Seeds were harvested from one plant for each RIL line, dried, stored at 10°C and were used for genotyping and phenotyping studies described below. These RILs thus represent lines advanced through single seed descent to F_8_. We genotyped 182 RILs while phenotyping was done only on 150 lines. Fewer RILs were used for phenotyping because 32 RILs did not germinate or had fewer seeds than needed for the current work.

### Phenotyping of parents and RILs post-germination

“N22,” “IR64” and 150 RILs were evaluated for germination at two temperatures 28 and 37°C for 4 days. The initial characterization of “N22” and “IR64” revealed a 4 days exposure of germinating seedlings to high temperature stress was enough to differentiate both shoot and root growth (Data not shown). Therefore, we used this time period to phenotype the RILs. The seeds for each line were dehusked and surface-sterilized by rinsing for 1 min with 70% (v/v) alcohol followed by 20 mL of 50% (v/v) bleach for 35 min. The seeds were then rinsed six times in sterile water and then allowed to imbibe at 28°C under complete darkness. The imbibed seeds were then divided into two sets each having 12 seeds per set in 100 mm × 15 mm petri plates lined with wet Whatman 2 filter paper. The two plates represented two blocks and 10 replicate seedlings per plate were used for data collection. The plates were covered with an aluminum foil wrap to ensure complete darkness. The sets of plates were incubated at 28°C (control) and 37°C (heat stress) for 4 days, respectively, using an incomplete block design where the screen was divided in 8 batches and the parents (“IR64” and “N22”) were included in each batch. After 4 days, shoot length and root length were measured on seedlings from both conditions using a ruler.

The growth of shoot and root under stress as percent of their respective controls was computed by the following formulae:

$$\begin{array}{lll} \text{SLPC} & = & {\left(\text{SLH}/\text{SLC}\right)}^{*}100\\ \text{RLPC} & = & {\left(\text{RLH}/\text{RLC}\right)}^{*}100 \end{array}$$

Where, SLPC = Shoot length under heat stress as percent of control, SLH = Shoot length under heat stress, SLC = Shoot length under control condition, RLPC = Root length as percent of control, RLH = Root length under heat stress, and RLC = Root length under control condition. SLPC and RLPC were calculated by using the mean root length and shoot length values for each RIL and the parents.

The broad sense heritability (H^2^) was computed using mean variance of the two parents over 8 batches as variance due to environment (V_E_) and the mean variance from RILs (V_P_) as the total phenotypic variance. Variance due to genotype (V_G_) was obtained by subtracting V_E_ from V_P_. The broad sense heritability was then computed as H^2^ = V_G_/V_P_.

All of the phenotypic data were tested for the goodness of fit to ascertain whether the data were normally distributed. SAS JMP 11.0, 2013 (SAS Institute Inc, Cary, NC, USA) was used for normality test based on Shapiro Wilk test (Shapiro and Wilk, 1965). Variances were calculated using SAS JMP software. The data on SLPC and RLPC were arcsine-transformed and confirmed for normality for use in QTL analysis.

### DNA extraction

A total of 182 RILs (F_8_) and the two parents “N22” and “IR64” were grown in the greenhouse for 1 month as described before. The leaf samples from individual lines were collected, frozen in liquid nitrogen and stored in −80°C freezer. To extract the genomic DNA, the leaf samples were ground into a powder using a plastic pestle with brief dipping of the microfuge tubes containing the samples in liquid nitrogen. The nuclear DNA was extracted using the CTAB method as described by Vallejos (2007) with minor modifications. One of the modifications was to use 0.3% (v/v) of β-mercaptoethanol in the extraction buffer instead of 1% and the other was washing the nuclear pellet after low speed centrifugation with nuclear suspension buffer. Briefly, after grinding the sample, 1 mL of sample suspension buffer containing 100 mM sodium acetate (pH 5.2), 25 mM EDTA (pH 8), 200 mM NaCl, and 10% (v/v) Triton X-100 was added followed by mixing using a vortex and then centrifuged at 200 *g* for 10 min. The supernatant was discarded while the pellet of nuclei was washed using 1 mL of nuclear resuspension buffer containing 900 mM Tris.HCL (pH 8.0) and 5 mM EDTA. The pellet was again centrifuged at 200 *g* for 10 min and thereafter the supernatant was discarded. The formed pellet was suspended in 300 μL of nuclear resuspension buffer containing 900 mM Tris.HCL pH 8.0 and 5 mM EDTA pH 8.0 with 5 μL of 10 mg/mL RNase A (Sigma Aldrich, Saint Louis, MO, USA**)** followed by mixing using a vortex. After a 10 min-incubation at 60°C, equal volume of 2X lysis buffer (200 mM Tris.HCl pH 7.8, 10 mM EDTA Na pH 8.0, 2.8 M NaCl, and 4% (wt/v) CTAB) was added to the sample tube and incubated at 65°C for 1 h. The sample was mixed by inverting every 5 min. Then, 450 μL of chloroform was added to extract the samples. About 600 μL of supernatant was transferred into new tube. The DNA was precipitated by adding 600 μL of isopropanol and incubating for 1 h. The pellet was washed with 76% (v/v) ethanol followed by a 100% ethanol wash. The air-dried DNA pellet was dissolved in 100 μL of 0.1X TE buffer (10 mM Tris-HCl, pH 8.0; 1 mM EDTA, pH 8.0.). The quality and quantity of the DNA were determined using gel electrophoresis and Nano drop (Epoch Microplate spectrophotometer, BioTek instrument Inc, Winooski, VT, USA), respectively.

### Library construction for GBS

The library preparation was done by Cornell University's Institute of Biotechnology's genomic diversity facility, Ithaca NY. About 100 ng of DNA from each of the 182 RILs and the two parents were digested using *ApeK*I. This restriction enzyme does not cut the most repetitive chromosomal regions and recognizes a 5 bp degenerate sequence (GCWGC), leaving a 3 bp overhang at the 5‘ end. This enzyme is optimal at 75°C, is not sensitive to *dam* and *dcm* methylation, but is sensitive to CpG methylation. To construct the library, the methods by Elshire et al. (2011) were adapted, where the two types of adaptors (oligonucleotides) with common (3′ *ApeK*I cut site) and barcode adaptors at 5′ end on both bottom and top strand were added into 96 well plate following dilution in 50 μM TE buffer. For annealing, the barcodes were incubated at thermocycler set at 95°C for 2 min, then down to 25°C at the rate of 0.1°C/s, and 25°C for 30 min. Both types of oligonucleotides and DNA samples were diluted, and then quantified with PicoGreen (Thermo Scientific, Waltham, MA, USA) to a concentration of 0.6 and 100 ng/ μL, respectively. Then equal amount of barcodes was added in a 96 well plate, dried and then 10 μL containing 100 ng DNA from each of the RILs and the two parental varieties were added each in a single well. The DNA samples and barcodes were then digested using *Ape*KI (New England Biolab, IpSwith, MA, USA) for 2 h, while plates were incubated in water bath set at 75°C. Following the 2 hours digestion, the adaptors were ligated to DNA sticky ends by T4 DNA ligase enzyme (30 μL) and then all samples (96 × 2 plates) were pooled together (5 μL each), purified and PCR-amplified. The components of PCR were 2 μL DNA fragments, 1X *Taq* Master mix, and 25 pmol of primers in overall volume of 50 μL. The PCR reaction was for 18 cycles of 72°C for 5 min, 98°C for 30 s, and 65°C for 30 s and extension at 72°C for 5 min. The libraries were then purified and size selected based on an automated gel electrophoresis. The fragment size, which was considered good, was 84 bp. These libraries were sequenced in two lanes in duplicates with Illumina HiSeq 2500 platform (Illumina, Inc., San Diego, CA, USA).

### Sequence analysis and SNP calling

To obtain the list of tags present in the raw sequences, reads with “Ns” and any mismatch in the barcode sequences were trimmed out to remain with reads for further analysis (Tassel, version 5.0). The above analysis generated a separate file containing sequence reads for each RIL in the population. The filtered sequence data were aligned to the reference genome of rice *indica* group with ID number GCA_000004655.2 assembled by the European Bioinformatics Institute (EBI), (Squizzato et al., 2015) using Burrows-Wheeler Alignment (bwa version 0.7.8-r455) software (Li and Durbin, 2009). The default edit of 4 per sequence and no *indels* within the last 5 bp of the reads' ends was applied for controlling the balance between SNPs and confidence alignment as well as prevention of gap penalty. Following alignment, GBS sequence data was processed into SNP genotypes using TASSEL version: 3.0.165: 2014 (Bradbury et al., 2007). To ensure only highly informative markers are retained, SNP marker data was filtered for minor allele frequency (0.1) and imputation of missing genotypes was performed by running Beagle's imputation algorithm using default parameters (Browning and Browning, 2016). The resulting dataset was converted to haplotype format in Tassel. All heterozygous calls were replaced as missing data and the resulting dataset was filtered again to keep the markers which were present in at least 90% of the RILs, and having more than 5% minor allele frequency.

### Construction of linkage map

Mapmarker 3.0 software (Lander et al., 1987) was used for sorting high quality SNP markers into 12 different groups and analysis of linkage between them. The JOIN HAPLOTYPES command was used to retain the most informative marker with unique recombination pattern from a haplotype bin. These markers were grouped into linkage groups using the GROUP command. A default linkage criteria of LOD 7 and 40 cM was used for grouping. The “informativeness criteria” were set at 2 cM and a minimum of 155 informative RILs. A multipoint criterion of 4 5 5 was used followed by the ORDER command to order the markers within a linkage group. This command allows building the mapping framework using a window size of 5 markers and a LOD threshold of 5. After establishing the initial map framework at LOD 5, the remaining markers were used to assign a position in the map at LOD 4. The markers that did not show enough linkage at LOD 4 were removed from the map. We used the TRY command and an LOD threshold of 3 to retain some of the excluded markers from the previous step. The resulting map was validated using the RIPPLE command with 5 markers at LOD 3. Any spuriously placed marker was removed and the map was tested again using RIPPLE command. Linkage maps were drawn using Mapchart (Voorrips, 2002). Chi square tests in the segregation of markers were applied to detect possible marker segregation distortion.

### QTL mapping

The following six quantitative phenotypic traits were used for quantitative trait locus/loci (QTL) detection: “shoot length under control condition” (SLC), “root length under control condition” (RLC), “shoot length under high temperature stress” (SLHT), “root length under high temperature stress” (RLHT), “shoot length under high temperature stress as percent of control” (SLPC) and “root length under high temperature stress as percent of control” (RLPC). The QTL analysis was conducted using composite interval mapping (CIM) and multiple interval mapping (MIM) algorithms as implemented in Windows QTL Cartographer Version 2.5_011 (Wang et al., 2012). CIM analysis was performed using forward stepwise regression and with 1000 permutations to detect the empirical threshold LOD and defining a QTL as significant. The analysis was conducted using 10 cM window size and 1 cM walk speed. The QTL from CIM analysis were used to build initial model for MIM (Kao et al., 1999; Zeng et al., 1999) analysis to detect additional QTL and epistatic interactions among them. New main effect QTL were searched with a minimum 5 cM distance between QTL and this step was repeated to update the model until no further QTL was identified. The significance of each QTL in the model was tested using likelihood ratio tests. The final MIM model was tested for improving LOD profiles and positions for each QTL and Bayesian Information Criteria (BIC) was used to select the best QTL models. The main effect QTL in the selected model was used to search for epistatic interactions among them. The MIM analysis was conducted separately for each trait. We defined the QTL effects as “Small” for *r*^2^ < 10%, “Intermediate” for *r*^2^ between 10–20%, and “Large” for *r*^2^ > 20%.

### Identification of putative candidate genes located in the QTL regions

*Oryza sativa* genomic sequence (version 7.0) in the Phytozome 12 (JGI, https://phytozome.jgi.doe.gov) database was scanned for predicted transcripts between markers flanking the QTL locations. Annotations for the transcripts were obtained from MSU rice genome annotation project (http://rice.plantbiology.msu.edu/) except for one region on chromosome 6 for which the annotations for the *indica* genome was used because this region significantly differed between *japonica* and *indica* genomes.

## Results

### Phenotyping the mapping population

**Figure 1:**
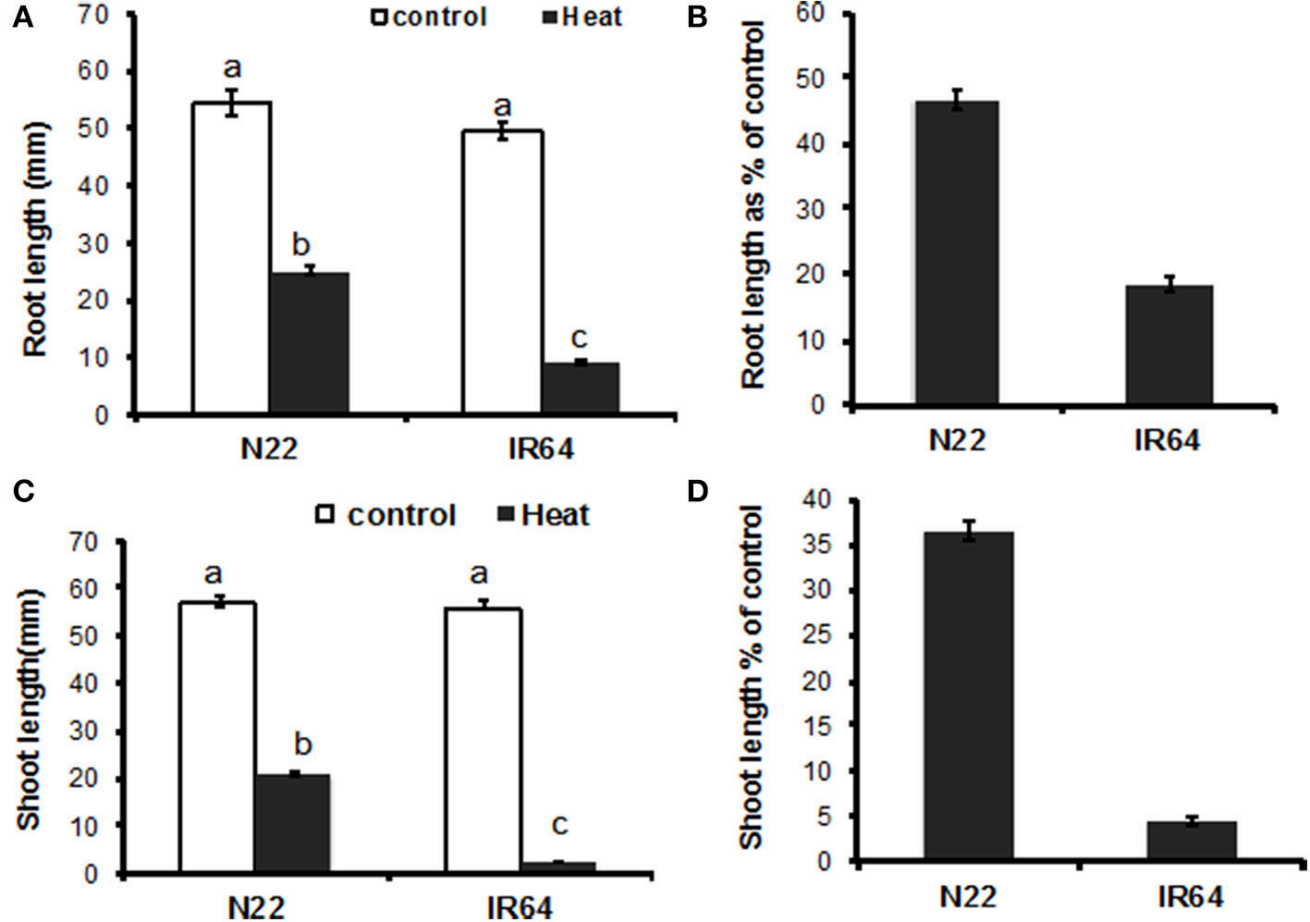
Heat stress tolerance during germination. Dehusked seeds of “N22” and “IR64” were allowed to germinate in petri plates in an incubator set at 28°C (control) for 24 h. The 1 day old germinating seedlings were kept at 28°C (control) or 37°C (heat stress) for 4 days under dark. Mean and standard error values (*n* = 20) are shown for **(A)** root length **(C)** shoot length, and **(B)** and **(D)** same data shown in **(A)** and **(C)** repectively, expressed as per cent of control. Means marked with different letters indicate significant differences at α = 0.05 using Duncan's multiple range test.

A comparative growth analysis of the parental genotypes “N22” and “IR64” showed no significant differences between them under mesothermic condition (28°C), and exposure to 37°C significantly (*p* = 0.05) inhibited both shoot and root growth of both genotypes (Figures 1A–D); however, the high temperature treatment had a significantly greater impact on “IR64” than on “N22” (Figures 1A–D). Shoot and root growth of “IR64” at 37°C represented only 4 and 18% of the sizes attained at mesothermic control temperature. In contrast, the high temperature treatment reduced “N22” shoots and roots only 36 and 46% of their control sizes, respectively (Figures 1B,D). The correlation analysis showed a weak positive correlation (*r* = 0.08) between the relative shoot and root growth in “IR64,” and a moderately positive correlation (*r* = 0.57) in “N22.”

**Figure 2:**
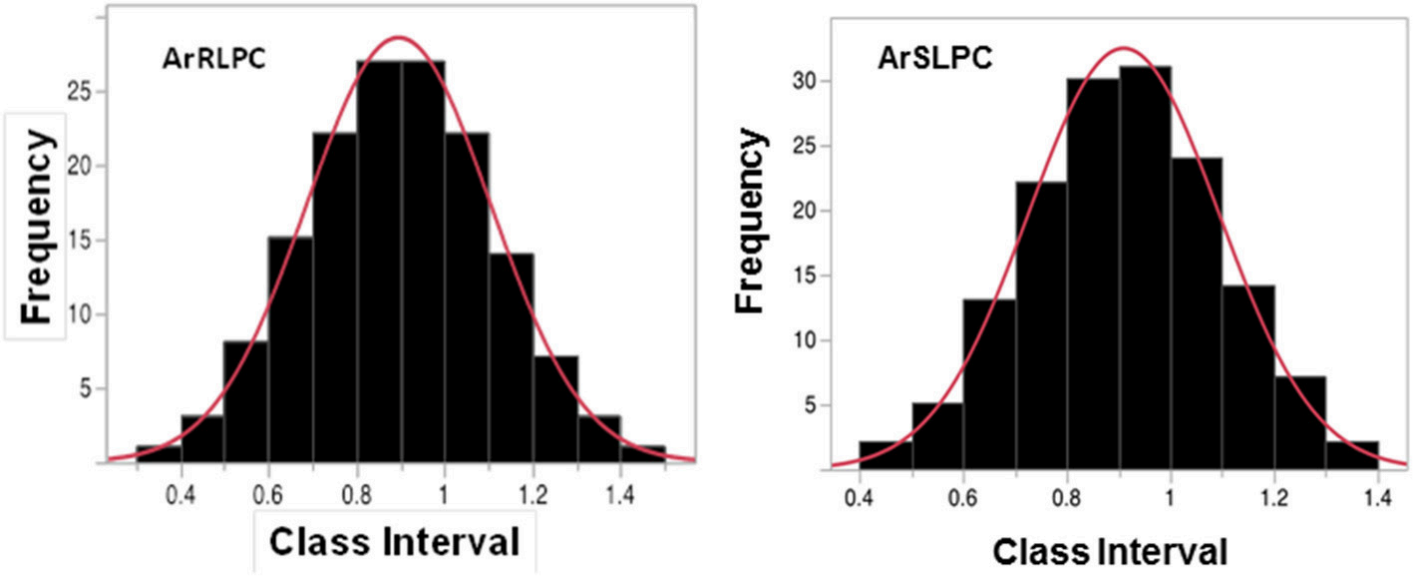
Frequency distribution of RLPC and SLPC data collected from 150 RILs derived from “N22” × “IR64,” exposed under control and heat stress conditions after arc-sine transformation. Bars represent the frequency values for different class intervals. JMP SAS normalized the data using arcsine transformation. The values for transformed RLPC for “N22” and “IR64” were 0.92 and 0.36, respectively and for SLPC were 0.74 and 0.30, respectively.

As significant phenotypic differences were observed between “N22” and “IR64,” we further evaluated the high temperature root and shoot growth responses of a recombinant inbred population derived from these two genotypes. A differential high temperature response was observed in the recombinant inbred population also but the relative effect varied extensively showing transgressive behavior. Some of the RILs performed better and others worse than either parent. Normality test indicated that both root and shoot growth of 150 RILs were not normally distributed with p-value of 0.0112 and 0.0252, respectively, and hence were normalized using arc-sine transformation (Figure 2). Seedling shoot and root growth had broad sense heritability values of 0.87 and 0.76, respectively under mesothermic condition and 0.96 and 0.86, respectively under heat stress.

### Genotyping-by-sequencing and SNP marker generation

The total number of raw reads was approximately 270.1 million in the first lane and 538.4 million in the second lane of Illumina HiSeq platform. The quality filtering of these raw reads, as explained in the methods, retained ~313.5 million high-quality barcoded reads. These high quality reads constituted about 20.8 million sequences with unique good-barcoded reads (“tags” in TASSEL) that showed further reduction after filtering for read depth criterion ≥3 (Remaining reads-−1,832,206). These 1,832,206 reads represent the 100% of the sequence output from this experiment. A total of 57.4% (1,050,884) reads have a unique alignment to the reference genome, while 8.7% (159,603) have multiple alignments. About 33.9% (621,719) of the total reads remain unaligned. The possible explanation for the lack of alignment of these reads includes, systematic errors during sequencing, origination of sequences from the highly repetitive DNA and errors during PCR amplification of the libraries. Following Tassel SNPcaller script, a total of 202,998 variant sites in the population were called, which were further filtered to remove the *indels* and SNPs with more than two allelic variants, resulting in a SNP dataset (*n* = 172,271). The dataset was filtered to keep the markers present in at least 90% of the RILS, and having more than 5% minor allele frequency. Then, a final filtering step was performed to retain only SNPs that showed polymorphism between the parental genotypes, which led to a total 4,074 high quality SNP markers for linkage analysis.

### Genotyping-by-sequencing-derived linkage map of “N22 × IR64”

The 4,074 high quality SNP markers belong to 1,638 recombinationally unique bins, while remaining 2,436 markers share the haplotype blocks with the most informative marker in recombination blocks (Table 1). Out of 1,638 recombinationally unique markers, linkage was detected among 689 marker loci at LOD threshold of 3 and these marker loci were spread across 15 linkage groups (Supplementary Figure 1). The markers from chromosome 1, 10, and 11 form two separate linkage groups at LOD threshold of 3. The two separate groups for chromosome 1 and 10 showed linkage at a lower LOD score, while no linkage was detected among the terminal markers on two chromosome 11 groups even at a lower threshold (Supplementary Figure 1). The linkage map covered the total distance of 1818.4. cM and varied in length among the linkage group from as low as 84.7 cM in chromosome 10 and 241.8 cM in chromosome 2 (Table 1, Supplementary Figure 1). In addition, the marker density was different among the 12 linkage groups that ranged from 18 markers in chromosome 10 and 113 in chromosome 1. The average distance between markers within a chromosome ranged from 2.13 cM in chromosome 2 to a maximum of 2.99 cM for chromosome 7 (Table 1). Data on the linkage map is provided in Supplemental File 1.

**Table 1:** Linkage analysis of molecular markers in a biparental mapping population derived from N22 × IR64.

**Linkage group**	**Total markers**	**Recombinationally unique markers**	**Mapped loci**	**cM**	**Avg. inter loci distance (cM)**
Lg-01 a,b	515	208	12, 70	44, 172.2	2.63
Lg-02	548	229	113	241.8	2.13
Lg-03	345	147	68	189.5	2.78
Lg-04	532	141	50	146.9	2.93
Lg-05	376	159	68	167.6	2.46
Lg-06	318	133	55	156.9	2.85
Lg-07	302	129	48	143.6	2.99
Lg-08	274	115	46	124.6	2.7
Lg-09	251	90	47	108.8	2.31
Lg-10 a,b	82	40	6, 12	12.9, 71.8	2.68
Lg-11 a,b	239	107	36, 6	82, 9.1	2.16
Lg-12	292	140	52	146.7	2.82
Total	4,074	1,638	689	1818.4	31.44
Average	339.5	136.5	57.4	121.2	2.62

*Single nucleotide polymorphism (SNP) markers were derived from genotyping-by-sequencing (GBS) procedure. Information on linkage groups, number of markers, distance covered (cM) by SNP markers, and average inter loci distance are shown. Lg—linkage group*.

A comparison of genetic map to the physical distance indicated a linear relationship with the genome of *Oryza sativa* var. *indica* (Supplementary Figure 2). The genetic to physical distance comparison identified Chr09 as a telocentric chromosome, confirming previous studies in *O. sativa* (Harushima et al., 1998). Further analysis of the rate of change of genetic vs. physical distance by taking a first derivative of linkage distance (cM) to map distance (Mb) ratio revealed distinct regions of high and low recombination across the rice genome. For example, the high recombination peaks were only present at the proximal ends for chromosomes 02, 05, 06, and 12 (Supplementary Figure 2). In contrast, some chromosomes (i.e., chromosomes 01, 04, and 07) exhibited high recombination regions in the middle part the chromosomes too. The peaks of recombination also differed in magnitude and number across different chromosomes (Supplementary Figure 2). For example, chromosomes 01, 02, 05, and 07 contained the highest recombination peaks with cM/Mbs values more than 400, while the other chromosomes including chromosomes 10 and 12, had peaks with much lower magnitude (Supplementary Figure 2).

### QTL mapping for shoot and root growth under control conditions

We used both the CIM and MIP to identify statistically significant QTL for shoot and root related traits (Tables 2, 3). Six QTL related to root growth under control condition were identified on chromosomes 1, 4, and 7 with 2–12% effect (Table 2). Two QTL were identified for “shoot length under control condition” both on chromosome 6 with 6% and 12% effects (Table 2). The nucleotide positions of these QTL and the number of annotated genes in those regions are shown in Supplementary Table 1.

**Table 2:** Description of quantitative trait loci (QTL) identified for seedling root length (RLC) and shoot length (SLC) under control condition, using composite interval mapping (CIM) and multiple interval mapping (MIM) analyses.

**Trait**	**QTL**	**Linkage group**	**Linked marker**	**Position (cM)**	**Marker interval**	**LOD**	**Additivity**	**Effect (%)**	**CIM threshold**
**RLC**									
	rlc1.1^*^	1b	S1_10221082	6.21	0–13.4	4.93	−4.12	9.5	3.42
	rlc1.2	1b	S1_30191377	89.91	80.4–94.3	4.67	4.17	4.9
	rlc4.1	4	S4_100099	0.01	0–4.5	5.95	−6.10	6.7
	rlc4.2	4	S4_1911293	8.81	5.4–11.2	5.62	8.02	3.7
	rlc4.3	4	S4_13167045	25.41	21.8–33.2	3.81	−6.01	2.1
	rlc7.1	7	S7_24934857	127.61	119.4–138.2	5.46	4.45	6.7
**SLC**									
	slc6.1	6	S6_9368784	54.21	41.2–59.5	3.96	0.05	6.4	3.23
	slc6.2	6	S6_32050861	156.71	145.2–156.9	5.28	−0.04	12.1

*The QTL with LOD score higher than the threshold values from CIM model, linked markers and marker intervals are presented here. The QTL positions and effects are presented from the MIM analysis. The negative additivity values represent the effect from “N22” parental allele and positive additivity values represent the effect from “IR64” parental allele, obtained as R^2^ values representing phenotypic variance that was explained by genetic variance in the MIM model*.

* *Detected in both CIM and MIM model*.

*Unmarked QTL were detected only using MIM model*.

**Table 3:** Description of quantitative trait loci (QTL) for root length under heat stress (RLHT), root length under heat stress as per cent of control (RLPC), shoot length under heat stress (SLHT) and shoot length under heat stress as per cent of control (SLPC), identified using composite interval mapping (CIM) and multiple interval mapping (MIM) analyses.

**Trait**	**QTL**	**Linkage group**	**Linked marker**	**Position (cM)**	**Marker interval**	**LOD**	**Additivity**	**Effect (%)**	**CIM threshold**
**RLHT**									
	rlht5.1^**^	5	S5_28173385	142.61	135.3–149.0	3.86	−0.04	20.4	3.03
**RLPC**									
	rlpc1.1	1a	S1_1860959	13.21	0–29.4	3.95	−0.04	5.2	3.12
	rlpc2.1	2	S2_861442	2.71	0–12.0	4.72	−0.05	7.5
	rlpc3.1^**^	3	S3_32129158	133.41	130.8–137.2	6.83	−0.08	8.6
	rlpc4.1^**^	4	S4_992878	4.41	0–7.5	6.21	−0.06	8.3
**SLHT**									
	slht3.1	3	S3_32129158	133.41	131.6–136.7	5.57	−0.06	7	3.02
	slht4.1	4	S4_992878	4.41	0–8.1	4.3	−0.05	5.3
	slht6.1	6	S6_32050861	156.71	142.4–156.9	4.18	−0.04	10.2
**SLPC**									
	slpc2.1	2	S2_861442	2.71	0–15.5	5.38	−0.12	19	3.12
	slpc4.1	4	S4_1357063	5.21	0–8.8	4.96	−0.07	8.1
	slpc3.1	3	S3_17056945	85.71	70.3–98.8	7.97	−0.05	8.6
	slpc5.1	5	S5_5758689	49.21	38.1–55.5	5.75	0.03	6.6
	slpc6.1	6	S6_32050861	156.71	135.0–156.9	7.75	−0.04	13.2
	slpc10.2	10b	S10_19121694	26.31	10.4–30.4	7.87	−0.20	17.1
	slpc10.3	10b	S10_19259739	27.31	11.7–32.2	5.69	0.13	17.9

*The QTL with LOD score higher than the threshold values from CIM model, linked marker and marker intervals are presented here. The QTL positions and effects are presented from the MIM analysis. The negative additivity values represent the effect from “N22” parental allele and positive additivity values represent the effect from “IR64” parental allele, obtained as R^2^ values representing phenotypic variance that was explained by genetic variance in the MIM model*.

** *Detected in both CIM and MIM models*.

*Unmarked QTL were detected using MIM model only*.

### QTL mapping for shoot and root growth under heat stress

We identified one QTL on linkage group 5 for “root length under heat stress (RLHT)” with 20% effect (Table 3). When “root length under heat stress” was expressed as per cent of control, four more QTL with effects varying between 5.2 and 8.6% were identified (Table 3). The QTL *rlht5.1* was a major QTL contributing 20% toward variation for the trait under study (Table 3). By examining the genomic sequence between the markers flanking the QTL, we identified multiple annotated genes in the QTL regions: 18 in *rlht5.1*, 62 in *rlpc1.1*, 10 in *rlpc2.1*, 87 in *rlpc3.1*, and 36 in *rlpc4.1* QTL regions (Table 4).

**Table 4:** Identification of transcripts between markers flanking quantitative trait loci (QTL) for root length under heat stress (RLHT), root length under heat stress as per cent of control (RLPC), shoot length under heat stress (SLHT) and shoot length under heat stress as per cent of control (SLPC).

**QTL**	**Nucleotide positions of the flanking markers**	**Length of the region (kb)**	**Number of gene models in the region**
rlht5.1	28173385 and 28287519	114.1	18
rlpc1.1	1860884 and 2313975	453	62
rlpc2.1 & slpc2.1	861429 and 911832	50.41	10
rlpc3.1	32129193 and 32630762	501.57	87
rlpc4.1 and slht4.1	992855 and 1327947	335.09	36
slpc4.1	1357155 and 1911385	54.23	40
slpc3.1	17057110 and 18163980	1,110	130
slpc5.1	5758641 and 6029552	270	26
slpc6.1 and slht6.1	32050861 and 32407949	357	87
slpc10.2 and slpc10.3	7677559 and 13443229	5,770	541

*The nucleotide positions in the genome sequence, the length of the chromosomal region scanned and the number of transcripts identified from Phytozome are shown*.

For “shoot length under heat stress (SLHT)” trait, three QTL were identified with 5.3%−10.2% effect and among these three, two *slht3.1* and *slht4.1* were at the same chromosomal locations as *rlpc3.1* and *rlpc4.1*, respectively (Table 3). When “shoot lengths under heat stress” were expressed as per cent of control (SLPC), additional seven QTL with effects from 6.6 to 19% were identified. Among them, *slpc2.1* was at the same chromosomal location as the root trait QTL *rlpc2.1*. Similarly, *slpc6.1* was at the same location as *slht6.1*. For shoot related traits by examining the genomic sequence between the markers flanking the QTL, we identified the following number of annotated genes: 40 in *slpc4.1*, 130 in *slpc3.1*, 38 in *slpc6.1/slht6.1*, and 26 in *slpc5.1* (Table 4). The region where two QTL for *slpc* were located on chromosome 10 had 541 annotated genes. Overall, our study revealed ten unique chromosomal regions with QTL for heat stress tolerance during post-germination growth (Tables 3, 4).

We identified genes potentially involved in stress tolerance by searching the annotations of genes within the detected QTL regions with an additional key word “stress” in the Web of Science^TM^ database (1900–2017, November 2017, Clarivate Analytics). In this analysis, transcripts annotated as “hypothetical protein” or “expressed protein” were not included. Reports connecting the gene with that annotation to stress, especially to abiotic stress in any plant system were identified based on these reports which are listed in Supplementary Table 2. This analysis narrowed 213 annotated genes as potential candidate genes for heat stress tolerance (Supplementary Table 2).

## Discussion

Previous studies identified the rice variety “N22” to be the most heat stress tolerant variety and have therefore used it to identify the chromosomal segments controlling heat stress tolerance during the reproductive stage (Jagadish et al., 2010a; Ye et al., 2012, 2015a,b; González-Schain et al., 2016; Shanmugavadivel et al., 2017). However, the major focus of these studies was stress imposed during reproductive stages. In the current study, we have focused on identifying QTL controlling vegetative stage heat stress tolerance of “N22” rice using a biparental mapping population.

Although many QTL have been identified, little is known about the potential causal genes controlling heat tolerance during reproductive stage (Ye et al., 2015a). According to Ye et al. (2015b), fine mapping of the major QTL for “spikelet fertility under heat stress” led to a chromosomal region with more than 200 genes. Shanmugavadivel et al. (2017) phenotyped a RIL population of 272 lines created between “N22” and “IR64,” for spikelet sterility and yield under heat stress and mapped multiple QTL using a linkage map with 824 SNP markers.

Taking advantages of the two rice varieties markedly differing in their tolerance to heat stress, the initial characterization of germinating “N22” and “IR64” seedlings showed differential responses to high temperature stress while the differences in growth of both shoot length and root length under normal conditions between the two varieties were not statistically significant (Figure 1).The exposure to 37°C significantly (*p* = 0.05) inhibited both shoot and root in both varieties than their respective controls. Our findings had similar trends with those previously reported about “N22”s tolerance to heat stress under reproductive stages (Chang-lan et al., 2005; Jagadish et al., 2010a,b; Bahuguna et al., 2015; Ye et al., 2015a,b). The differential responses in both shoot and root growth under stress suggest possible unique mechanisms in response to high temperature stress in “N22.” The differences enabled us to screen 150 RILs generated from a cross between “N22” and “IR64” for root and shoot growth under heat stress post germination (Figure 2), which were later, used for QTL mapping.

We used a simple and rapid screening procedure to evaluate heat stress tolerance during post-germination growth using controlled environmental conditions. For each RIL and treatment condition, we used 12 high quality seeds (based on plumpness and free from disease) that were first dehusked, surface-sterilized and imbibed in sterile water for 1 day prior to placing them on sterile wet paper under complete darkness at either 28 or 37°C conditions. Hence, the data on coleoptile length and root length reflect the heterotrophic growth processes under normoxic conditions during phase III of the germination and post-germination growth (Bewley, 1997). During this phase, we can expect active mobilization of stored reserves, processes promoting cell elongation and cell division and active protein synthesis. Therefore, our measurements should reflect differential tolerance of these processes to high temperature stress between “N22” and “IR64.”

We employed GBS, a low-cost genotyping method that utilizes next-generation sequencing (NGS) technologies to identify SNPs (Elshire et al., 2011). Our initial set of SNP markers is larger than that of Spindel et al. (2013) who had pioneered the GBS protocol to obtain more than 30,000 markers using a bi-parental mapping population of rice between “IR64” and “Azucena.” However, in our study, we selected highly informative polymorphic markers following imputation of the GBS data (Spindel et al., 2013) with a minimum sequencing depth criterion of 3, which reduced the total number of markers to 4,074. However, of these, 1,638 markers were recombinationally unique and 2,436 markers were in haplotype blocks. We used 689 markers to develop the presented map (Supplementary Figure 1). In our study, the distribution of the markers was different in each linkage group but each linkage group had more than 100 markers except linkage groups 9 and 10 (Table 1). One reason for the discrepancies may be due to sequencing errors and more markers being in haplotype blocks.

Our linkage map allowed us to precisely identify multiple QTL, related to “root growth under stress as percent of its respective control” and root growth under heat stress. Using root growth and shoot growth under control and heat stress conditions, we have identified eight QTL for seedling growth (Table 2) and ten QTL for heat stress tolerance (Table 3). QTL *rlc1.1*, on chromosome 1 and *slc6.2*, on chromosome 6 were the major QTL for root and shoot growth during germination, (Table 2). Previous studies on seedling vigor in rice have identified QTL for coleoptile length and root length (Xie et al., 2014; Eizenga et al., 2016; Lee et al., 2017; Singh et al., 2017), on chromosomes 1,3, and 6 using different mapping populations.

Our study identified ten QTL for heat stress tolerance on chromosomes 1, 2, 3,4,5,6, and 10 (Table 3) similar to studies which identified multiple QTL for heat tolerance during flowering scored by spikelet sterility (Jagadish et al., 2010a; Ye et al., 2012, 2015a,b; Shanmugavadivel et al., 2017). In Ye et al. (2012)'s study, two major QTL were identified: one on the fourth chromosome with favorable alleles by “N22” and another on chromosome 1 with favorable alleles by “IR64.” The QTL identified for heat stress tolerance at post-germination stage in the current study do not overlap with any of the QTL identified for heat stress tolerance during the reproductive stage in studies by Ye et al. (2012, 2015a,b) and Shanmugavadivel et al. (2017). The lack of overlap between QTL found for reproductive stage stress tolerance and vegetative stage tolerance could reflect differences in the stress tolerance mechanisms or the methods employed to phenotype the plants or both. Among the QTL identified in our study, *rlht5.1, slht6.1*/*slpc6.1, slpc2.1, slpc10.2*, and *slpc10.3* were major QTL with >10% effect (Table 3). QTL identified were in regions where the recombination rate derived by comparing linkage distance to map distance ranged from 0.75 to 13.8 (Supplemental File 1; Table 4), suggesting the potential for fine mapping some of the QTL. To our knowledge, this is the first report of major QTL for heat stress tolerance during vegetative stage in rice. When the length of the QTL region between flanking markers were calculated, for two QTL the distances between flanking markers were less than 60 kb (Table 4), limiting possible annotated genes in those regions to about 50 or less (Table 4).

We do not have a well annotated genome sequence for “*aus*” type “N22” landrace. Hence, we used transcript annotations for *O. sativa japonica* genome to identify the transcripts encoded by genes in the QTL regions. We systematically tested whether the gene models in the QTL regions were implicated in stress tolerance in any plant system. Although, we omitted genes annotated as “hypothetical protein” or “expressed protein,” it is to be emphasized that some of the genes coding for hypothetical proteins are likely novel candidate genes for heat stress tolerance and need to be tested in future experiments. Our analysis revealed that 213 annotated genes found between boundary markers for QTL for heat stress tolerance (Supplementary Table 2) were implicated in stress tolerance in previous studies. For QTL for differential growth of roots or shoots under high temperature stress, the potential candidate genes were found in four functional categories namely (a) gene regulation and signaling (i.e., DNA-binding domain containing proteins, transcription factors and kinases), (b) protein protection and processing (cold acclimation protein, heat shock proteins, remorin family protein, subtilisins, protease inhibitor protein, and F-box domain proteins), (c) defense and disease resistance (DNA repair protein, defensins and pentatricopeptide protein) and (d) metabolism (3-ketoacyl CoA synthase, lipid phosphatase, fatty acid hydrolase, P450 and oxidoreductases, decarboxylase and glutathione sulfotransferase) (Supplementary Table 2). For example, *rlpc1.1* had genes encoding a fatty acid hydroxylase, calmodulin binding protein, calmodulin-related Ca sensor, hsp20/alpha crystalline family heat shock protein, lipid phosphatase, leucine rich protein, B3 DNA binding domain protein, WD domain containing protein, a peptide transporter and a MYB family transcription factor (Supplementary Table 2). Among these, the function of heat shock proteins in heat stress tolerance is well known (Bita and Gerats, 2013). It is possible that any one or more than one of these could be candidate genes for differential heat stress tolerance between “IR64” and “N22.” Our results are consistent with early studies on drought stress induced genes in “N22” compared to “IR64” in vegetative seedlings that showed that heat shock proteins and chaperonins, zinc-finger proteins, and certain defense related proteins were expressed significantly more in “N22” than in “IR64” (Gorantla et al., 2007). A study on SNP discovery compared “N22” with “IR64” and identified differential expression between these two varieties for genes in the categories of macromolecular modification, phosphorylation and phosphate metabolic processes (Jain et al., 2014).

Ye et al. (2015b) reported that the 1.2 Mb region of the *qHTSF4.1* had 24 cell wall-associated receptor kinase genes and six rapid alkalinization factor (RALF) genes and speculated that cell wall-associated kinase (WAK) genes may be candidate genes as they were known in cell expansion and tolerance to biotic and abiotic stress and chilling. The short list of annotated genes we have identified included annotations for receptor kinases: *rlpc1.1* region had two OsWAK receptor-like cytoplasmic kinases, *slpc4.1* region had five genes for kinases and *slpc3.1* had five kinases and *slpc10.2* region had two kinases. Gene annotations of annexin (*slpc3.1*, Supplementary Table 2) and receptor-like kinases are shared with annotations for candidate genes identified by Shanmugavadivel et al. (2017) for reproductive stage heat stress tolerance. Together these relationships between the gene annotations in QTL for reproductive stage heat stress tolerance and annotations of genes in QTL for vegetative stage tolerance suggest that even if the chromosomal regions controlling the stress tolerance trait under the two developmental stages are different, some of the proteins involved in stress tolerance could mechanistically be overlapping. Future functional studies on mutants affected in the identified potential candidate genes will confirm the relevance of these genes for high temperature stress tolerance of “N22.” The SNP markers at the boundary of the QTL will be valuable in marker-assisted selection for vegetative stage heat stress tolerance in rice breeding research. In conclusion, our study has revealed multiple QTL and associated SNP markers for high temperature stress tolerance in rice. In particular, our results in part explain the nature of high temperature stress tolerance found in “N22” variety. This is the first study to link potential candidate genes to vegetative stage tolerance to high temperature stress in rice.

## Author contributions

BR, CV, and PK designed the study and secured funding, CY and SJ contributed key resources. NK conducted the experiments. JS performed the bioinformatics analyses and all the authors participated in writing and revising the manuscript. NK and JS have contributed equally to this work.

### Conflict of interest statement

The authors declare that the research was conducted in the absence of any commercial or financial relationships that could be construed as a potential conflict of interest.

## Supplementary material

Supplementary Figure 1 The genetic map of rice constructed with polymorphic SNP markers showing linkage groups 1, 2, 3, 4, 5, 6, 7, 8, 9, 10, 11, and 12. The chromosomal location is marked on the left and the marker positions on the genome sequence on the right. The graphical representation was done using MapChart software.

Supplementary Figure 2 Scatter plot showing the relationship between linkage map distances (cM) and physical distances (Mb) among 12 chromosomes indicated by red lines with red squares representing the markers mapped for each of the 12 chromosomes. The recombination rate (cM/Mb) is shown using black lines.

Supplemental Table 1 Identification of transcripts between markers flanking quantitative trait loci (QTL) for root length under control condition (rlc), and shoot length under control condition (slc), shown in Table 2.

Supplementary Table 2 Annotations of transcripts in the QTL regions that have potential connections to stress adaptation.
