# Supplementary figures and images for: Heat Stress Tolerance in Rice (Oryza sativa L.): Identification of Quantitative Trait Loci and Candidate Genes for Seedling Growth Under Heat Stress
Source: Front Plant Sci. 2018 Nov 1;9:1578. doi: 10.3389/fpls.2018.01578 (PMC6221968; doi:10.3389/fpls.2018.01578)

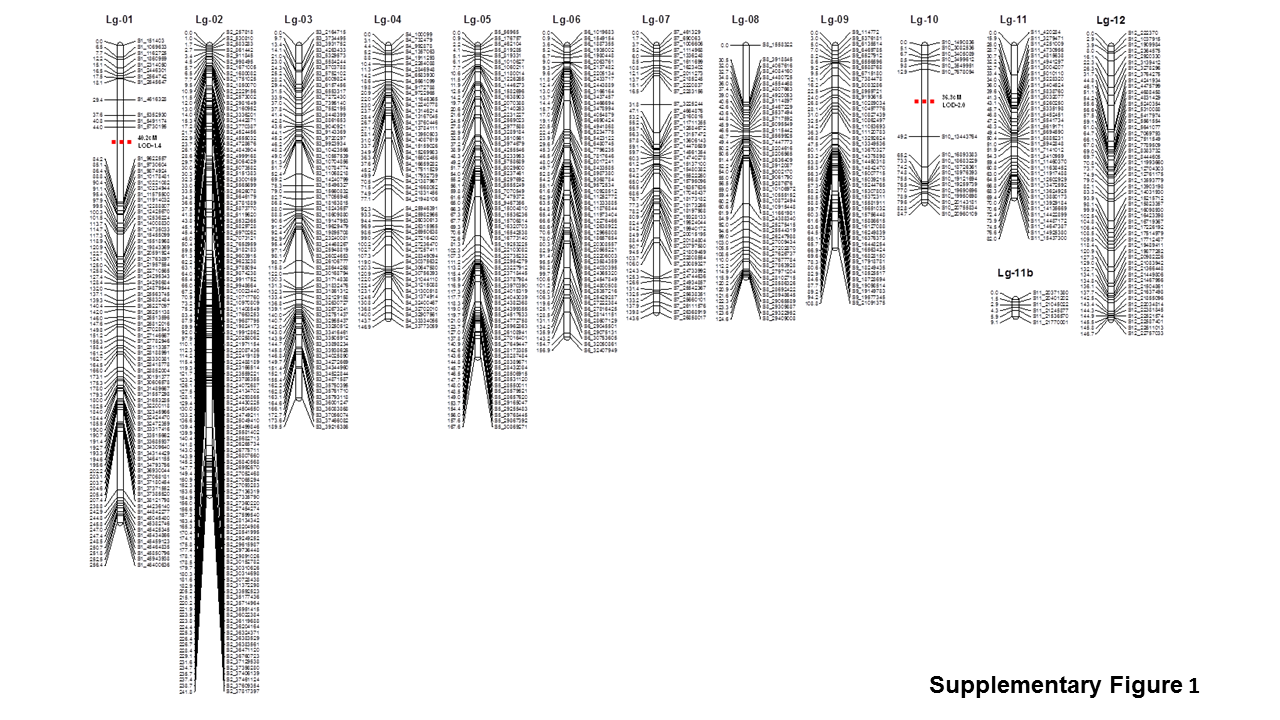

Supplement: Supplementary Figure 1 — The genetic map of rice constructed with polymorphic SNP markers showing linkage groups 1, 2, 3, 4, 5, 6, 7, 8, 9, 10, 11, and 12. The chromosomal location is marked on the left and the marker positions on the genome sequence on the right. The graphical representation was done using MapChart software. [file Image_1.TIF]

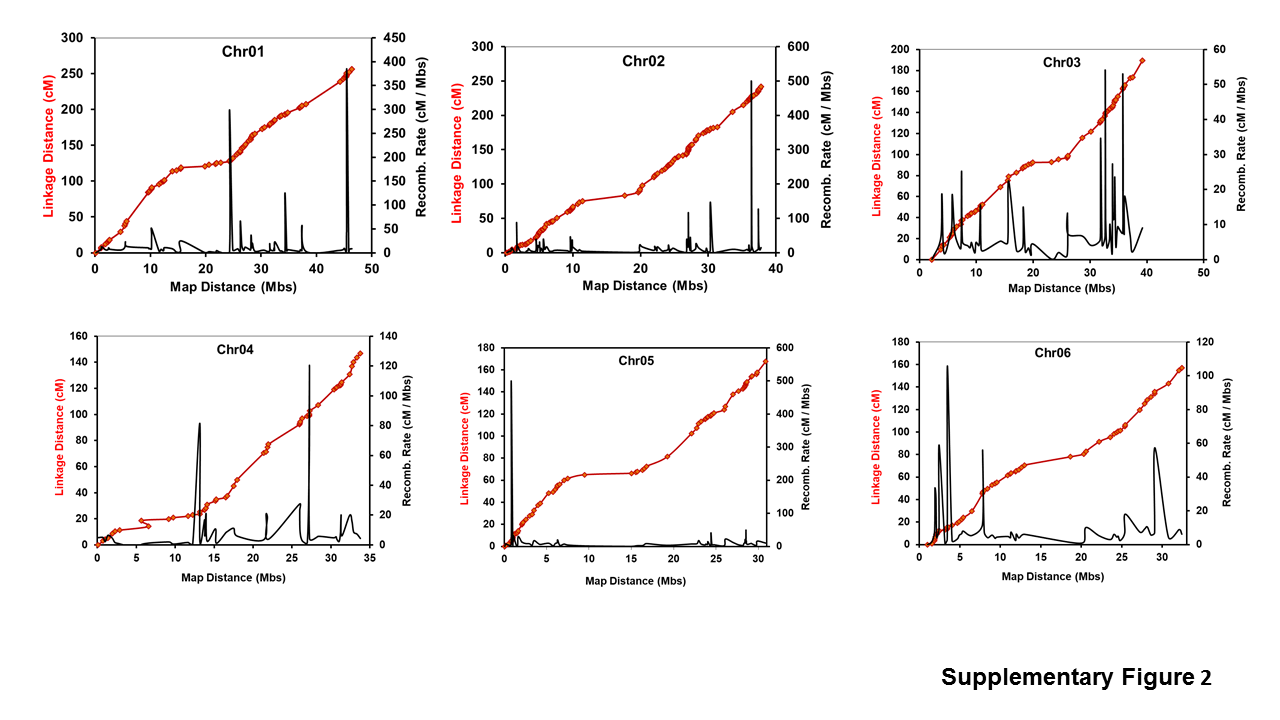

Supplement: Supplementary file 2 [file Image_2.TIF]

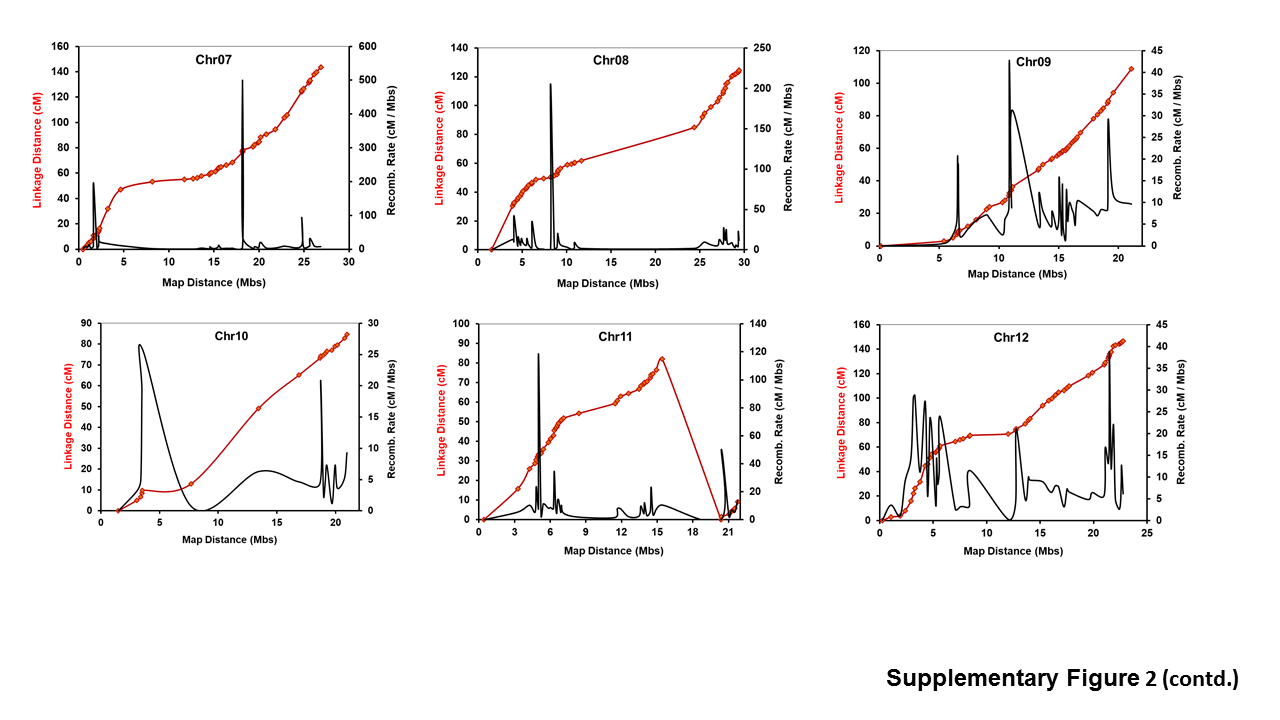

Supplement: Supplementary Figure 2 — Scatter plot showing the relationship between linkage map distances (cM) and physical distances (Mb) among 12 chromosomes indicated by red lines with red squares representing the markers mapped for each of the 12 chromosomes. The recombination rate (cM/Mb) is shown using black lines. [file Image_3.TIF]
