# Supplementary material for: Heat Stress Tolerance in Rice (Oryza sativa L.): Identification of Quantitative Trait Loci and Candidate Genes for Seedling Growth Under Heat Stress
Source: Front Plant Sci. 2018 Nov 1;9:1578. doi: 10.3389/fpls.2018.01578 (PMC6221968; doi:10.3389/fpls.2018.01578)
Supplement: Supplemental Table 1 — Identification of transcripts between markers flanking quantitative trait loci (QTL) for root length under control condition (rlc), and shoot length under control condition (slc), shown in Table 2. [file Table_1.DOCX]

**Supplemental Table 1.** Identification of transcripts between markers flanking quantitative trait loci (QTL) for root length under control condition (rlc), and shoot length under control condition (slc), shown in Table 2. The nucleotide positions in the genome sequence, the length of the chromosomal region scanned and the number of transcripts identified from Phytozome are shown.

| **QTL** | **Nucleotide positions of the flanking markers** | **Length of the region (kb)** | **Number of transcripts in the region** |
| --- | --- | --- | --- |
| rlc1.1 | 10178481 and 10234944 | 56.47 | 6 |
| rlc1.2 | 30191377 and 30606578 | 415.2 | 60 |
| rlc4.1 | 100099 and 732479 | 632.38 | 77 |
| rlc4.2 | 1911293 and 2264088 | 352.7 | 22 |
| rlc4.3 | 13167045 and 13760446 | 593.4 | 41 |
| rlc7.1 | 24934857 and 25542367 | 607.3 | 86 |
| slc6.1 | 8987380 and 9572534 | 585.2 | 58 |
| slc6.2 | 32050861 and 32407949. | 357 | 87 |
|  |  |  |  |

**Supplementary Table 2.** Annotations of transcripts in the QTL regions that have potential connections to stress adaptation. Key word searches were done using ‘Web of Science’ database using the gene annotations combined with the words ‘stress’ and ‘plant’ to identify possible reports in *Oryza sativa* or other plants. In cases where multiple reports regarding the gene’s role in stress adaptation are available, only one report or a review on the topic is referred here. Annotations were abbreviated for simplicity (e.g. ‘domain-containing protein’ as ‘domain protein’, and the omission of words such as ‘putative’ and organellar location information ‘chloroplast precursor’). Bibliography listing the references for this table is presented as a supplemental file.

| **QTL** | **Gene ID** | **Annotation (Abbreviated)** | **Reference connecting the gene to stress** |
| --- | --- | --- | --- |
| rlht5.1 | Os05g49150 | Eukaryotic translation initiation factor 3 | Singh et al. (2013) |
|  | Os05g49170 | Cold acclimation protein WCOR413 | Zhang et al. (2010) |
|  | Os05g49200 | Aspartic proteinase oryzasin-1 | Choudhary et al (2015) |
|  | Os05g49220 | GTP-binding protein | Xu and Cai (2014) |
|  | Os05g49250 | hhH-GPD superfamily DNA repair protein | Nota et al. (2015) |
|  | Os05g49280 | GATA Zn finger domain protein | Zhang et al. (2015) |
|  | Os05g49290 | 3-ketoacyl-CoA synthase | Lee et al., (2009) |
| rlpc1.1 | Os01g04260 | fatty acid hydroxylase | Nagano et al. (2012) |
|  | Os01g04280 | calmodulin binding protein | Liu and Yu (2017) |
|  | Os01g04330 | calmodulin-related calcium sensor |  |
|  | Os01g04340 | hsp20/alpha crystallin family | Sarkar et al. (2009) |
|  | Os01g04350 | hsp20/alpha crystallin family | Sarkar et al. (2009) |
|  | Os01g04360 | hsp20/alpha crystallin family | Sarkar et al. (2009) |
|  | Os01g04370 | hsp20/alpha crystallin family | Sarkar et al. (2009) |
|  | Os01g04380 | hsp20/alpha crystallin family | Sarkar et al. (2009) |
|  | Os01g04409 | OsWAK receptor-like cytoplasmic kinase | Zhang et al. (2005) |
|  | Os01g04450 | OsWAK receptor-like cytoplasmic kinase | Zhang et al. (2005) |
|  | Os01g04460 | Ser/Thr protein kinase | Zhang et al. (2013) |
|  | Os01g04490 | Ser/Thr protein kinase | Zhang et al. (2013) |
|  | Os01g04520 | Ser/Thr protein kinase | Zhang et al. (2013) |
|  | Os01g04540 | Ser/Thr protein kinase | Zhang et al. (2013) |
|  | Os01g04570 | Ser/Thr protein kinase | Zhang et al. (2013) |
|  | Os01g04580 | Ser/Thr protein kinase | Zhang et al. (2013) |
|  | Os01g04650 | PB1 domain protein | Long et al. (2013) |
|  | Os01g04660 | Lipid phosphatase | McLoughlin and Testerink (2013) |
|  | Os01g04720 | Leucine rich protein | Van Der Does et al. (2017) |
|  | Os01g04750 | B3 DNA binding domain protein | Min et al (2014) |
|  | Os01g04800 | B3 DNA binding domain protein | Min et al (2014) |
|  | Os01g04870 | WD, G-beta repeat domain containing protein | Islas-Flores et al (2015) |
|  | Os01g04930 | MYB family transcription factor | Smita et al (2015) |
|  | Os01g04950 | Peptide transporter | Ouyang et al (2010) |
| rlpc2.1 | Os02g02480 | Basic helix-loop DNA binding domain protein | Xu et al (2017) |
|  | Os02g02490 | Phytosulfokine receptor precursor | Sandve et al (2008) |
|  | Os02g02500 | Remorin family protein | Yue et al (2014) |
|  | Os02g02530 | Signal peptide peptidase domain protein | Baldwin et al (2014) |
| rlpc3.1 | Os03g56440 | F-box domain containing protein | Jain et al (2007) |
|  | Os03g56450 | F-box domain containing protein | Jain et al (2007) |
|  | Os03g56460 | Glucose-6-phosphate isomerase | Yin et al (2017) |
|  | Os03g56470 | Protein kinase family protein | Lafarge et al (2017) |
|  | Os03g56510 | F-box domain containing protein | Jain et al (2007) |
|  | Os03g56540 | Heat shock protein DNAJ | Mishra and Grover (2016) |
|  | Os03g56580 | No Apical Meristem protein | Gao et al (2010) |
|  | Os03g56660 | Calmodulin binding protein | Virdi et al (2015) |
|  | Os03g56670 | Photosystem I reaction center subunit | Sonoike (1996) |
|  | Os03g56682 | Defensin and Defensin-like family | De Coninck et al (2010) |
|  | Os03g56790 | Actin-depolymerizing factor | Huang et al (2012) |
|  | Os03g56820 | Fatty acid hydroxylase | Nagano et al (2012) |
|  | Os03g56840 | GTP binding protein | Ruanjaichon et al (2014) |
|  | Os03g56850 | Pentatricopeptide | Wu et al (2016) |
|  | Os03g56869 | Ribose 5 phosphate isomerase | Xiong et al (2009) |
|  | Os03g56944 | Lys-rich arabinogalactan protein | Schultz et al (2002) |
|  | Os03g56950 | Phytochrome-interacting factor | Xie et al (2017) |
|  | Os03g56960 | PPR repeat domain containing protein | Tan et al (2014) |
|  | Os03g57040 | Clathrin adaptor complex small chain domain protein | Singh et al (2016) |
|  | Os03g57070 | Amine oxidase-related protein | Wimalasekera et al (2011) |
|  | Os03g57080 | Phospholipase PLA IIIA/PLP7 |  |
|  | Os03g57120 | Ferredoxin-NADP reductase | Kozuleva et al (2016) |
|  | Os03g57160 | Zinc ion binding protein | Tomas et al (2014) |
|  | Os03g57190 | TCP family transcription factor | Almeida et al (2017) |
|  | Os03g57200 | Glutathione S-transferase | Wu et al (2017) |
|  | Os03g57210 | Glycine-rich protein | Ortega-Amaro et al (2015) |
| rlpc4.1 &slht4.1 | Os04g02640 | 3-ketoacyl-CoA synthase | Lee et al (2009) |
|  | Os04g02720 | Potassium channel | Lebaudy et al (2007) |
|  | Os04g02754 | Amidase family protein | Liu et al (2015) |
|  | Os04g02780 | Amidase family protein | Liu et al (2015) |
|  | Os04g02820 | Elongation factor | Momcilovic et al (2016) |
|  | Os04g02850 | Pentatricopeptide | Liu et al (2016) |
|  | Os04g02860 | Disease resistance protein | Bao et al (2014) |
|  | Os04g02870 | RNA recognition protein | Cheng et al (2017) |
|  | Os04g02920 | Leucine rich repeat family | Van Der Does et al (2017) |
|  | Os04g02960 | Subtilisin homologue | Golldack et al (2003) |
|  | Os04g02970 | Subtilisin homologue | Golldack et al (2003) |
|  | Os04g02980 | Subtilisin homologue | Golldack et al (2003) |
|  | Os04g02990 | Subtilisin homologue | Golldack et al (2003) |
|  | Os04g03050 | Subtilisin homologue | Golldack et al (2003) |
|  | Os04g03080 | Subtilisin homologue | Golldack et al (2003) |
|  | Os04g03100 | Subtilisin homologue | Golldack et al (2003) |
| slpc4.1 | Os04g03210 | Receptor kinase | Kang et al (2017) |
|  | Os04g03320 | Jacalin-like lectin domain protein | Ma et al (2010) |
|  | Os04g03360 | Jacalin-like lectin domain protein | Ma et al (2010) |
|  | Os04g03370 | Cys-rich receptor-like protein kinase 41 | Xu et al (2016) |
|  | Os04g03530 | Cys-rich receptor-like protein kinase 37 | Xu et al (2016) |
|  | Os04g03579 | Protein kinase | Bundo and Coca (2017) |
|  | Os04g03710 | Subtilisin homologue OsSub36 | Golldack et al (2003) |
|  | Os04g03796 | Subtilisin homologue OsSub37 | Golldack et al (2003) |
|  | Os04g03810 | Subtilisin homologue OsSub38 | Golldack et al (2003) |
|  | Os04g03830 | OsWAK receptor-like protein kinase | Kang et al (2017) |
|  | Os04g03850 | Subtilisin homologue OsSub39 | Golldack et al (2003) |
|  | Os04g03870 | Cytochrome P450 | Tamiru et al (2015) |
|  | Os04g03890 | Cytochrome P450 | Tamiru et al (2015) |
|  | Os04g03980 | Flavin monooxygenase | Lee et al (2012) |
|  | Os04g04060 | Dynamin family protein | Banaei-Asl et al (2015) |
| slpc3.1 | Os03g29970 | Histone-like transcription factor | Manimaran et al (2017) |
|  | Os03g29980 | ACT domain protein | Hsieh and Goodman (2002) |
|  | Os03g30050 | Protein kinase family protein | Bundo and Coca (2017) |
|  | Os03g30130 | Phospholipase | Li et al (2017) |
|  | Os03g30220 | Mitochondrial import inner membrane translocase subunit Tim17 | Wojtyla et al (2013) |
|  | Os03g30230 | Mitochondrial import inner membrane translocase subunit Tim17 | Wojtyla et al (2013) |
|  | Os03g30400 | Early light-induced protein | Zhuo et al (2013) |
|  | Os03g30420 | Cytochrome P450 | Tamiru et al (2015) |
|  | Os03g30470 | Chitinase family protein | Kashyap and Deswal (2017) |
|  | Os03g30550 | RNA-binding protein homologue | Park et al (2017) |
|  | Os03g30570 | DUF1675 domain protein | Cui et al (2016) |
|  | Os03g30610 | Ubiquinone oxidoreductase | Nwugo and Huerta (2011) |
|  | Os03g30800 | Fumoarylacetoacetate hydrolase family | Dashevskaya et al (2013) |
|  | Os03g30870 | Progesterone 5-beta reductase | Herl et al (2006) |
|  | Os03g30910 | Disease resistance protein RPM1 | Bao et al (2014) |
|  | Os03g30920 | F-box domain protein OsFBX95 | Jain et al (2007) |
|  | Os03g30950 | Acyl desaturase | Klickkenberg et al (2014) |
|  | Os03g31000 | Protein kinase | Bundo and Coca (2017) |
|  | Os03g31070 | Protein kinase | Bundo and Coca (2017) |
|  | Os03g31180 | Diacylglycerol kinase | Arisz et al (2013) |
|  | Os03g31210 | UDP-glucose 6-dehydrogenase | Dubey et al (2003) |
|  | Os03g31230 | MYB family transcription factor | Deeba et al (2017) |
|  | Os03g31240 | C2-H2 zinc finger protein | Yin et al (2017) |
|  | Os03g31260 | Cys-rich receptor-like protein kinase 28 | Xu et al (2016) |
|  | Os03g31290 | Membrane-anchored ubiquitin-fold protein | Park et al (2011) |
|  | Os03g31300 | ClpB Chaperone protein | Singh et al (2012) |
|  | Os03g31310 | Circadian Clock coupling factor related | Mikkelesen and Thomashow (2009) |
|  | Os03g31320 | RING zinc finger protein | Yin et al (2017) |
|  | Os03g31340 | Protein transport protein SEC61 | Jang et al (2005) |
|  | Os03g31360 | Glutelin | Liu et al (2015) |
|  | Os03g31370 | Gly-rich protein | Fujino et al (2014) |
|  | Os03g31400 | U-box domain protein | Wang et al (2016) |
|  | Os03g31430 | Terpene synthase | Lee et al (2015) |
|  | Os03g31480 | Expansin precursor | Chen et al (2017) |
|  | Os03g31510 | Cys proteinase inhibitor 8 | Chojnacka et al (2015) |
|  | Os03g31550 | Aldehyde oxidase | Srivastava et al (2017) |
|  | Os03g31570 | Pro-rich family protein | Liu et al (2015) |
|  | Os03g31594 | JmjC Domain protein | Li et al (2015) |
|  | Os03g31630 | Subtilisin homologue OsSub29 | Golldack et al (2013) |
|  | Os03g31679 | Annexin A7 | Qiao et al (2015) |
|  | Os03g31690 | GCN5-related N acetyltransferase |  |
|  | Os03g31750 | Pyruvate phosphate dikinase | Hyskova et al (2014) |
| slpc5.1 | Os05g10580 | Cullin family domain protein (F box) | Jain et al (2007) |
|  | Os05g10620 | No Apical Meristem protein | Gao et al (2010) |
|  | Os05g10650 | 6-Phosphofructokinase | Mustroph et al (2013) |
|  | Os05g10670 | Zn finger CCCH type family protein | Jan et al (2013) |
|  | Os05g10690 | Myb Transcription factor | Deeba et al (2017) |
|  | Os05g10730 | ABC transporter | Matsuda et al (2016) |
|  | Os05g10770 | Jumonji Transcription factor | Hou et al (2016) |
|  | Os05g10780 | Aminotransferase | You et al (2012) |
|  | Os05g10840 | Calmodulin-binding protein | Yue et al (2015) |
| slpc10.2 | Os10g22860 | Receptor kinase | Ouyang et al (2010) |
|  | Os10g22890 | Receptor kinase | Ouyang et al (2010) |
|  | Os10g22920 | Leu-rich repeat N terminal domain protein | Kadota and Shirasu (2012) |
|  | Os10g22930 | Disease resistance protein |  |
|  | Os10g22950 | Calmodulin-binding protein | Yue et al (2015) |
|  | Os10g22960 | Hydrolase | Opassiri et al (2007) |
|  | Os10g22980 | Leu-rich repeat domain protein | Kadota and Shirasu (2012) |
|  | Os10g23050 | Helix-loop-helix DNA binding protein | Wang et al (2003) |
|  | Os10g23090 | Homeobox associated Leu zipper | Chew et al (2013) |
|  | Os10g23100 | Ras-related protein | Zang et al (2010) |
|  | Os10g23120 | Pyridoxamine-5’-phosphate oxidase | Gonzalez et al (2007) |
|  | Os10g23130 | Cytochrome P450 88A1 | Wang et al (2012) |
|  | Os10g23160 | Ent-kaurenoic acid oxidase | Wang et al (2012) |
|  | Os10g23180 | Cytochrome P450 | Wang et al (2012) |
|  | Os10g23230 | AT hook-containing MAR binding | Morisawa et al (2000) |
|  | Os10g23830 | PPR-repeat domain containing | Asano et al (2013) |
|  | Os10g23900 | Decarboxylase | Roy and Wu (2001) |
|  | Os10g24010 | F-box domain containing | Jain et al (2007) |
|  | Os10g24090 | Glucosyltransferase | Li et al (2015) |
|  | Os10g24150 | Zn knuckle family | Kohli et al (2014) |

**References cited in Supplementary Table 2.**

Almeida, D.M., Gregorio, G.B., Oliveira, M.M., Saibo, N.J.M. (2017) Five novel transcription factors as potential regulators of OsNHX1 gene expression in a salt tolerant rice genotype. Plant Mol. Biol. 93: 61-77.

Arisz, S.A., Wijk, R., Roels, W., Zhu, J.K., Haring, M.A., Munnik, T (2013) Rapid phosphatidic acid accumulation in response to low temperature stress in Arabidopsis is generated through diacylglycerol kinase. Front. Plant Sci. doi:10.3389/fpls.2013.00001.

Baldwin, M., Russo, C., Li, X., Chishti, A.H. (2014) *Plasmodium falciparum* signal peptide peptidase cleaves malaria heat shock protein 101 (HSP101). Implications for gametocytogenesis. Biochem. Biophys. Res. Commun. 450: 1427-1432.

Bao, F., Huang, X., Zhu, C., Zhang, X., Li, X., Yang, S (2014) *Arabidopsis* HSP90 protein modulates RPP4-mediated temperature-dependent cell death and defense responses. New Phytol. 202: 1320-1334.

Bundo, M., Coca, M (2017) Calcium-dependent protein kinase OsCPK10 mediates both drought tolerance and blast disease resistance in rice plants. J. Exp. Bot. 68: 2963-2975.

Chen, Y., Han, Y., Kong, X., Kang, H., Ren, Y., Wang, W (2017) Ecotopic expression of wheat expansin gene *TaEXPA2* improved the salt tolerance of transgenic tobacco by regulating Na+/K+ and antioxidant competence. Physiol. Plant. 159: 161-177.

Cheng, C., Wang, Z., Yuan, B., Li, X (2017) RBM25 mediates abiotic responses in plants. Front. Plant Sci. 8:292.

Chojnacka, M., Szewinska, J., Mielecki, M., Nykiel, M., Imai, R., Bielawski, W., Orzechowski, S (2015) A triticale water-deficit-inducible phytocystatin inhibits endogenous cysteine proteinases *in vitro*. J. Plant Physiol. 174: 161-165.

Choudhary, M., Jayanand, Parida, J.C. (2015) Transcriptional profiling in pearl millet (*Pennisetum glaucum* L.R. Br.) for identification of differentially expressed drought responsive genes. Physiol. Mol, Biol. Plants 21: 187-196.

Cui, Y., Wang, M., Zhou, H., Li, M., Huang, L., Yin, X., Zhao, G., Lin, F., Xia, X., Xu, G (2016) OsSGL, a novel DUF1645 domain-containing protein, confers enhanced drought tolerance in transgenic rice and *Arabidopsis*. Front. Plant Sci. 7:2001.

Dashevskaya, S., Horn, R., Chudobova, I., Schillberg, S., Velez, S.M.R., Capell, T., Christou, P (2013) Abscisic acid and the herbicide safener cyprosulfamide cooperatively enhance abitoic stress tolerance in rice. Mol Breeding 32: 463-484.

De Coninck, B.M.A., Sels, J., Venmans, E., Thys, W., Goderis, I.J.W.M., Carron, D., Delaure, Cammue, B.P.A., De Bolle, M.F.C., Mathys, J. (2010) *Arabidopsis thaliana* plant defensin AtPDF1.1 is involved in the plant response to biotic stress. New Phytol. 187: 1075-1088.

Deeba, F., Sultana, T., Javaid, B., Mahmood, T., Naqvi, S.M.S. (2017) Molecular characterization of a MYB protein from *Oryza sativa* for its role in abiotic stress tolerance. Brazilian Arch. Biol. Technol. 60: e17160352.

Dubey, H., Bhatia, G., Pasha, S., Grover, A. (2003) Proteome maps of flood-tolerant FR 13A and flood-sensitive IR 54 rice types depicting proteins associated with O_2_-deprivation stress and recovery regimes. Current Sci. 84: 416-426.

Fan, T., Wang, R., Xiang, Y., An, L., Cao, S. (2016) Heat stress induces actin cytoskeletal reorganization and transcript profiles of vegetative profilins and actin depolymerizing factors (ADFs) in *Arabidopsis*. Acta Physiol. Plant. 38: 37.

Fujino, K., Obara, M., Sato, K (2014) Diversification of the plant-specific hybrid glycine-rich protein (HyGRP) genes in cereals. Front. Plant Sci. 5: Article 489.

Gao, F., Xiong, A., Peng, R., Jin, X., Xu, J., Chen, J., Yao, Q. (2010) OsNAC52, a rice NAC transcription factor, potentially responds to ABA and confers drought tolerance in transgenic plants. Plant Cell Tiss. Organ Cult. 100: 255-262.

Golldack, D., Vera, P., Dietz., K.J. (2003) Expression of subtilisin-like serine proteases in *Arabidopsis thaliana* is cell-specific and responds to jasmonic acid and heavy metals with developmental differences. Physiol. Plant. 118: 64-73.

Herl, V., Fischer, G., Muller-Uri, F., Kreis, W (2006) Molecular cloning and heterologous expression of progesterone 5-beta-reductase from *Digitalis lanata* Ehrh. Phytochem. 67: 225-231.

Hsieh, M.H., Goodman, H.M. (2002) Molecular characterization of a novel gene family encoding ACT domain repeat proteins in *Arabidopsis*. Plant Physiol. 130: 1797-1806.

Huang, Y., Huang, W., Hong, C., Lur, H., Chang, M (2012) Comprehensive analysis of differentially expressed rice actin depolymerizing factor gene family and heterologous overexpression of OsADF3 confers *Arabidopsis thaliana* drought tolerance. Rice 5:33.

Hyskova, V.D., Miedzinska, L., Dobra, J., Vankova, R., Ryslava, H (2014) Phosphoenolpyruvate carboxylase, NADP-malic enzyme, and pyruvate, phosphate dikinase are involved in the acclimation of *Nicotiana tabacum* L. to drought stress. J. Plant Physiol. 171: 19-25.

Islas-Flores, T., Rahman, A., Ullah, H., Villanueva, M.A. (2015) The receptor for activated C kinase in plant signaling: Tale of a promiscuous little molecule. Front. Plant Sci. 6: 1090.

Jain, M., Nijhawan, A., Arora, R., Agarwal, P., Ray, S., Sharma, P., Kapoor, S., Tyagi, A.K., Khurana, J.P. (2007) F-Box proteins in rice. Genome-wide analysis, classification, temporal and spatial gene expression during panicle and seed development, and regulation by light and abiotic stress. Plant Physiol. 143: 1467-1483.

Jang, C.S., Lee, T.G., Kim, J.Y., Park, J.H., Kim, D.S., Park, J.H., Seo, Y.W. (2015) The molecular characterization of a cDNA encoding the putative membrane protein, *HvSec61α,* expressed during early stage of barley kernel development. Plant Sci. 168: 233-239.

Kashyap, P., Deswal, R (2017) A novel class I chitinase from *Hippophae rhamnoides*: Indications for participating in ICE-CBF cold stress signaling pathway. Plant Sci. 258: 62-70.

Kim, D.S., Jeun, Y., Hwang, B.K. (2014) The pepper patatin-like phospholipase CaPLP1 functions in plant cell death and defense signaling. Plant Mol. Biol. 84: 329-344.

Klinkenberg, J., Faist, H., Saupe, S., Lambertz, S., Krischke, M., Stingl, N., Fekete, A., Mueller, M.J., Feussner, I., Hedrich, R., Deeken, R. (2014) Two fatty acid desaturases, STEAROYL-ACYL CARRIER PROTEIN DELTA-9 DESATURASE6 and FATTY ACID DESATURASE3, are involved in drought and hypoxia stress signaling in Arabidopsis crown galls. Plant Physiol. 164: 570-583.

Kozuleva, M., Goss, T., Twachtmann, M., Rudi, K., Trapka, J., Selinski, J., Ivanov, B., Garapati, P., Steinhoff, H., Hase, T., Scheive, R., Klare, J.P., Hanke, G.T. (2016). Ferredoxin:NADP(H) oxidoreductase abundance and location influences redox poise and stress tolerance. Plant Physiol. 172: 1480-1493.

Lafarge, T., Bueno, C., Frouin, J., Jacquin, L., Courtois, B., Ahmadi, N (2017) Genome-wide association analysis for heat tolerance at flowering detected a large set of genes involved in adaptation to thermal and other stresses. PLoS ONE 12: e0171254.

Lebaudy, A., Vavasseur, A., Hosy, E., Dreyer, I., Leonhardt, N., Thibaud, J.B., Very, A.A., Simonneau, T., Sentenac, H (2008) Plant adaptation to fluctuating environment and biomass production are strongly dependent on guard cell potassium channels. Proc. Natl. Acad. Sci. U.S.A. 105: 5271-5276.

Lee, G.W., Lee, S., Chung, M., Jeong, Y.S., Chung, B.Y. (2015) Rice terpene synthase 20 (OsTPS20) plays an important role in producing terpene volatiles in response to abiotic stresses. Protoplasma 252: 997-1007.

Lee, K., Back, K (2017) Overexpression of rice serotonin *N*-acetyltransferase 1 in transgenic rice plants confers resistance to cadmium and senescence and increases grain yield. J. Pineal Res. DOI:10.1111/jpi.12392.

Lee, S., Jung, S., Go, Y., Kim, H., Kim, J., Cho, H., Park, O.K., Suh, M. (2009) Two Arabidopsis 3-ketoacyl CoA synthase genes, KCS20 and KCS2/DAISY, are functionally reductant in cuticular wax and root suberin biosynthesis, but differentially controlled by osmotic stress. The Plant J. 60: 462-475.

Lee, S., Jung, S., Go, Y., Kim, H., Kim, J., Cho, H., Park, O.K., Suh, M. (2009) Two Arabidopsis 3-ketoacyl CoA synthase genes, KCS20 and KCS2/DAISY, are functionally redundant in cuticular wax and root suberin biosynthesis, but differentially controlled by osmotic stress. Plant J. 60: 462-475.

Li, J., Yu, C., Wu, H., Luo, Z., Ouyang, B., Cui, L., Zhang, J., Ye, Z. (2015) Knockdown of a JmjC domain-containing gene JMJ524 confers altered gibberellin responses by transcriptional regulation of GRAS protein lacking the DELLA domain genes in tomato. J. Exp. Bot. 66: 1413-1426.

Li, L., Wang, F., Yan, P., Jing, W. Zhang, C., Kudla, J., Zhang, W (2017) A phosphoinositide-specific phospholipase C pathway elicits stress-induced Ca2+ signals and confers salt tolerance to rice. New Phytol. 214: 1172-1187.

Liu, A., Yu, Y., Li, R., Duan, X., Zhu, D., Sun, X., Duanmu, H., Zhu, Y (2015) A novel hybrid proline-rich type gene GsEARLI17 from *Glycine soja* participated in leaf cuticle synthesis and plant tolerance to salt and alkali stresses. Plant Cell Tiss. Organ Cult. 121: 633-646.

Liu, J.M., Zhao, J.Y., Lu, P.P., Chen, M., Guo, C.H., Xu, Z.S., Ma, Y.Z. (2016) The E-subgroup pentatricopeptide repeat protein family in *Arabidopsis thaliana* and confirmation of the responsiveness PPR96 to abiotic stresses. Front. Plant Sci. 7:1825.

Liu, S., Xu, H., Wang, W., Li, N., Wang, W., Moller, I.M., Song, S. (2015) A proteomic analysis of rice seed germination as affected by high temperature and ABA treatment. Physiol. Plant. 154: 142-161.

Liu, X.P., Yu, L.X (2017) Genome-wide association mapping of loci associated with plant growth and forage production under salt stress in Alfalfa (*Medicago sativa* L.). Front. Plant Sci. 8:853.

Long, R., Yang, Q., Kang, J., Chao, Y., Wang, P., Wu, M., Qin, Z., Sun, Y. (2012) Molecular cloning and characterization of a novel stress responsive gene in alfalfa. Biol. Plant. 56: 43-49.

Manimaran, P., Reddy, V., Moin, M., Reddy, R.M., Yugandhar, P., Mohanraj, S.S., Balachandaran, S.M., Kirti S.P.B. (2017) Activation-tagging in indica rice identifies a novel transcription factor subunit, NF-YC13 associated with salt tolerance. Sci. Rep. 7: 9341.

McLoughlin, F., Testerink, C (2013) Phosphatidic acid, a versatile water-stress signal in roots. Front. Plant Sci. 4: article 525.

Min, H., Zheng, J., Wang, J. (2014) Maize *ZmRAV1* contributes to salt and osmotic stress tolerance in transgenic Arabidopsis. J. Plant Biol. 57:28-42.

Mishra, R.C., Grover, A (2016) ClpB/Hsp100 proteins and heat stress tolerance in plants. Critical Rev. Biotechnol. 36: 862-874.

Momcilovic, I., Pantelic, D., Zdravkovic-Korac, S., Oljaca, J., Rudic, J., Fu, J (2016) Heat-induced accumulation of protein synthesis elongation factor 1A implies an important role in heat tolerance in potato. Planta 244: 671-679.

Nagano, M., Takahara, K., Fujimoto, M., Tsutusmi, N., Uchimiya, H., Kawai-Yamada, M (2012) Arabidopsis sphingolipid fatty acid 2-hydroxylases (AtFAH1 and AtFAH2) are functionally differentiated in fatty acid 2-hydroxylastion and stress responses. Plant Physiol. 159: 1138-1148.

Nota, F., Cambiagno, D.A., Ribone, P., Alvarez, M.E. (2015) Expression and function of AtMBD4L, the single gene encoding the nuclear DNA glycosylase MBD4L in Arabidopsis. Plant Sci. 235: 122-129.

Nuruzzaman, M., Sharoni, A.M., Satoh, K., Kumar, A., Leung, H., Kikuchi, S. (2014) Comparative transcriptome profiles of the WRKY gene family under control, hormone-treated, and drought conditions in near-isogenic rice lines reveal differential, tissue specific gene activation. J Plant Physiol. 171: 2-13.

Nwugo, C.C., Huerta, A.J. (2011) The effect of silicon on the leaf proteome of rice (*Oryza sativa* L.) plants under cadmium stress. J. Proteome Res. 10: 518-528.

Ortega-Amaro, M.A., Rodriguez-Hernandez, A.A., Kessler, M.R., Hernandz-Lucero, E., Rosales-Mendoza, S., Ibanez-Salazar, A., Delgado-Sanchez, P., Jimenez-Bremont, J.F. (2015) Overexpression of AtGRDP2, a novel glycine-rich domain protein, accelerates plant growth and improves stress tolerance. Front. Plant Sci. 5: article 782.

Ouyang, J., Cai, Z., Xia, K., Wang, Y., Duan, J., Zhang, M. (2010) Identification and analysis of eight peptide transporter homologs in rice. Plant Sci. 179: 374-382.

Ouyang, Y., Chen, J., Xie, W., Wang, L., Zhang, Q. (2009) Comprehensive sequence and expression profile analysis of Hsp20 gene family in rice. Plant Mol. Biol. 70: 341-357.

Park, H.J., Park, H.C., Lee, S.Y., Bohnert, H.J., Yun, D.J. (2011) Ubiquitin and ubiquitin-like modifiers in plants. J. Plant Biol. 54: 275-285.

Park, Y., Choi, M.J., Park, S.J., Kang, H (2017) Three zinc-finger RNA-binding proteins in cabbage (*Brassica rapa*) play diverse roles in seed germination and plant growth under normal and abiotic stress conditions. Physiol. Plant. 159: 93-106.

Qiao, B., Zhang, Q., Liu, D., Wang, H., Yin, J., Wang, R., He, M., Cui, M., Shang, Z., Wang, D., Zhu, Z (2015) A calcium-binding protein, rice annexin *OsANN1*, enhances heat stress tolerance by modulating the production of H_2_O_2_. J. Exp. Bot. 66: 5853-5866.

Ruanjaichon, V., Sangsrakru, D., Kamolsukyunyong, W., Siangliw, M., Toojinda, T., Tragoonrung, S., Vanavichit, A. (2004). Small GTP-binding protein gene is associated with QTL for submergence tolerance in rice. Russian J. Plant Physiol. 51: 648-657.

Sandve, S.R., Rudi, H., Asp, T., Rognli, O.A. (2008) Tracking the evolution of a cold stress associated gene family in cold tolerant grasses. BMC Evo. Biol. 8: 245.

Sarkar, N.K., Kim, Y., Grover, A (2009) Rice sHsp genes: genomic organization and expression profiling under stress and development. BMC Genomics 10: 393.

Schultz, C.J., Rumsewicz, M.P., Johnson, K.L., Jones, B.J., Gaspar, Y.M., Bacic, A (2002). Using genomic resources to guide research directions. The arabinogalactan protein gene family as a test case. Plant Physiol. 129: 1448-1464.

Singh, A., Mittal, D., Lavania, D., Agarwal, M., Mishra, R.C., Grover, A. (2012) OsHsfA2c and OsHsfB4b are involved in the transcriptional regulation of cytoplasmic *OsClpB* (*Hsp100*) gene in rice (*Oryza sativa* L.). Cell Stress Chapero. 17: 243-254.

Singh, B., Chauhan, H., Khurana, J.P., Khurana, P., Singh, P. (2013) Evidence for the role of wheat eukaryotic translation initiation factor 3 subunit g (TaelF3g) in abiotic stress tolerance. Gene 532: 177-185.

Singh, D., Yadav, N.S., Tiwari, V., Agarwal, P.K., Jha, B. (2016) A SNARE-like superfamily protein SbSLSP from the halophyte *Salicornia brachiata* confers salt and drought tolerance by maintaining membrane stability, K+/Na+ ratio, and antioxidant machinery. Front. Plant Sci. 7:737.

Smita, S., Katiyar, A., Chinnusamy, V., Pandey, D.M., Bansal, K.C. (2015) Transcriptional regulatory network analysis of MYB transcription factor family genes in rice. Front. Plant Sci. 6: 1157.

Sonoike, K (1996) Photoinhibition of photosystem I: Its physiological significance in the chilling sensitivity of plants. Plant Cell Physiol. 37: 239-247.

Srivastava, S., Brychkova, G., Yarmolinsky, D., Soltabayeva, A., Samani, T., Sagi, M.( 2017) Aldehyde oxidase 4 plays a critical role in delaying silique senescence by catalyzing aldehyde detoxification. Plant Physiol. (in press).

Tamiru, M., Undan, J.R., Takagi, H., Abe, A., Yoshida, K., Undan, J.Q., Natsume, S., Uemura, A., Saitoh, H., Matsumura, H., Urasaki, N., Yokota, T., Terauchi, R (2015) A cytochrome P450, OsDSS1, is involved in growth and drought stress responses in rice (*Oryza sativa* L.). Plant Mol. Biol. 88: 85-99.

Tan, J.J., Tan, Z.H., Wu, F.Q., Sheng, P.K., Heng, Y.Q., Wang, X.H., Ren, Y.L., Wang, J.L., Guo, X.P., Zhang, X., Cheng, Z.J., Jiang, L., Liu, X.M., Wang, H.Y., Wan, J.M. (2014) A novel chloroplast-localized pentatricopeptide repeat protein involved in splicing affects chloroplast development and abiotic stress response in rice. Mol. Plant. 7: 1329-1349.

Tomas, M., Pagani, M.A., Andreo, C.S., Capdevila, M., Bofill, R., Atrian, S (2014) His-containing plant metallothioneins: comparative study of divalent metal-ion binding by plant MT3 and MT4 isoforms. J. Biol. Inorg. Chem. 19: 1149-1164.

Van der Does, D., Boutrot, F., Engelsdorf, T., Rhodes, J., McKenna, J.F., Vernhettes, S. et al. (2017) The Arabidopsis leucine-rich repeat receptor kinase MIK2/LRR-KISS connects cell wall integrity sensing, root growth and response to abiotic and biotic stresses. PLoS Genet 13: e1006832.

Van der Does, D., Boutrot, F., Engelsdorf, T., Rhodes, J., McKenna, J.F., Vernhettes, S., et al. (2017) The Arabidopsis leucine-rich repeat receptor kinase MIK2/LRR-KISS connects cell wall integrity sensing, root growth and response to abiotic and biotic stresses. PLoS Genet. 13: e1006832.

Virdi, A.S., Singh, S., Singh, P. (2015) Abiotic stress responses in plants: roles of calmodulin-regulated proteins. Front. Plant Sci. 6: 809.

Wang, N., Liu, Y., Cong, Y., Wang, T., Zhong, X., Yang, S., Li, Y., Gai, J. (2016) Genome-wide identification of soybean U-box E3 ubiquitin ligases and roles of GmPUB8 in negative regulation of drought stress response in Arabidopsis. Plant Cell Physiol. 57: 1189-1209.

Wimalasekera, R., Villar, C., Begum, T., Scherer, G.F.E. (2011) Copper Amine Oxidase1 (CuAO1) of Arabidopsis thaliana contributes to abscisic acid- and polyamine-induced nitric oxide biosynthesis and abscisic acid signal transduction. Mol. Plant 4: 663-678.

Wojtyla, L., Kosmala, A., Garnczarska, M (2013) Lupine embryo axes under salinity stress. II. Mitochondrial proteome response. Acta Physiol. Plant. 35: 2383-2392.

Wu, L., Wu, J., Liu, Y., Gong, X., Xu, J., Lin, D., Dong, Y. (2016). The rice pentatricopeptide repeat gene *TCD10* is needed for chloroplast development under cold stress. Rice 9: 67.

Wu, L.B., Ueda, Y., Lai, S., Frei, M (2017) Shoot tolerance mechanisms to iron toxicity in rice (*Oryza sativa* L.). Plant Cell Environ. 40: 570-584.

Xie, Y., Liu, Y., Wang, H., Ma, X., Wang, B., Wu, G., Wang, H (2017) Phytochrome-interacting factors directly suppress *MIR156* expression to enhance shade-avoidance syndrome in Arabidopsis. Nature Commun. 8: 348.

Xiong, Y., DeFraia, C., Williams, D., Zhang, X., Mou, Z (2009) Deficiency in a cytosolic ribose-5-phosphate isomerase causes chloroplast dysfunction, late flowering and premature cell death in Arabidopsis. Physiol. Plant. 137: 249-263.

Xu, P., Cai, W. (2014) RAN1 is involved in plant cold resistance and development in rice (*Oryza sativa*). J Exp Bot 65: 3277-3287.

Xu, X., Liu, X., He, X., Xu, L., Huang, Y., Shao, H., Zhang, D., Tang, B., Ma, H. (2017) The soybean basic helix-loop-helix transcription factor ORG3-like enhances cadmium tolerance via increased iron and reduced cadmium uptake and transport from roots to shoots. Front. Plant Sci. 8:1098.

Xu, X., Yu, T., Xu, R., Shi, Y., Lin, X., Xu, Q., Qi, X., Weng, Y., Chen, X. (2016) Fine mapping of a dominantly inherited powdery mildew resistance major-effect QTL, *Pm1.1*, in cucumber identifies a 41.1 kb region containing two tandemly arrayed cystein-rich receptor-like protein kinase genes. Theor. Appl. Genet. 129: 507-516.

Yin, M., Wang, Y., Zhang, L., Li, J., Quan, W., Yang, L., Wang, Q., Chan, Z (2017) The Arabidopsis Cys2/His2 zinc finger transcription factor ZAT18 is a positive regulator of plant tolerance to drought stress. J. Exp. Bot. 68: 2991-3005.

Yin, X., Hiraga, S., Hajika, M., Nishimura, M., Komatsu, S. (2017) Transcriptomic analysis reveals the flooding tolerant mechanism in flooding tolerant line and abscisic acid treated soybean. Plant Mol. Biol. 93: 479-496.

Yue, J., Li, C., Liu, Y., Yu, J. (2014) A remorin gene SiREM6, the target gene of SiARDP, from Foxtail millet (*Setaria italica*) promotes high salt tolerance in transgenic Arabidopsis. PLoS ONE 9:e100772.

Zhang, C., Hou, Y., Hao, Q., Chen, H., Chen, L., Yuan, S., Shan, Z., Zhang, X., Yang, Z., Qiu, D., Zhou, X., Huang, W. (2015) Genome-wide survey of the soybean GATA transcription factor gene family and expression analysis under low nitrogen stress. PLOS One 10:e0125174.

Zhang, H., Liu, W., Wan, L., Li, F., Dai, L., Li, D., Zhang, Z., Huang, R (2010). Functional analyses of ethylene response factor JERF3 with the aim of improving tolerance to drought and osmotic stress in transgenic rice. Transgenic Res. 19: 809-818.

Zhang, S., Chen, C., Li, L., Meng, L., Singh, J., Jiang, N., Deng, X., He, Z., Lemaux, P. (2005) Evolutionary expansion, gene structure, and expression of the rice wall-associated kinase gene family. Plant Physiol. 139: 1107-1124.

Zhang, Y., Wang, X., Li, Y., Wu, L., Zhou, H., Zhang, G., Ma, Z. (2013) Ectopic expression of a novel Ser/Thr protein kinase from cotton (*Gossypium barbadense*), enhances resistance to *Verticillium dahlia* infection and oxidative stress in Arabidopsis. Plant Cell Rep. 32: 1703-1713.

Zhuo, C., Cai, J., Guo, Z (2013) Overexpression of early light-induced protein (ELIP) gene from *Medicago sativa* ssp. *falcata* increases tole
